# No Evidence for a Second Evolutionary Stratum during the Early Evolution of Mammalian Sex Chromosomes

**DOI:** 10.1371/journal.pone.0045488

**Published:** 2012-10-19

**Authors:** Yukako Katsura, Yoko Satta

**Affiliations:** Department of Evolutionary Study of Biosystems, The Graduate University for Advanced Studies (Sokendai), Hayama, Kanagawa, Japan; North Carolina State University, United States of America

## Abstract

Mammalian sex chromosomes originated from a pair of autosomes, and homologous genes on the sex chromosomes (gametologs) differentiated through recombination arrest between the chromosomes. It was hypothesized that this differentiation in eutherians took place in a stepwise fashion and left a footprint on the X chromosome termed “evolutionary strata.” The evolutionary stratum hypothesis claims that strata 1 and 2 (which correspond to the first two steps of chromosomal differentiation) were generated in the stem lineage of Theria or before the divergence between eutherians and marsupials. However, this prediction relied solely on the molecular clock hypothesis between pairs of human gametologs, and molecular evolution of marsupial sex chromosomal genes has not yet been investigated. In this study, we analyzed the following 7 pairs of marsupial gametologs, together with their eutherian orthologs that reside in stratum 1 or 2: *SOX3/SRY*, *RBMX/Y*, *RPS4X/Y*, *HSFX/Y*, *XKRX/Y*, *SMCX/Y* (*KDM5C/D*, *JARID1C/D*), and *UBE1X/Y* (*UBA1/UBA1Y*). Phylogenetic analyses and estimated divergence time of these gametologs reveal that they all differentiated at the same time in the therian ancestor. We have also provided strong evidence for gene conversion that occurred in the 3′ region of the eutherian stratum 2 genes (*SMCX/Y* and *UBE1X/Y*). The results of the present study show that (1) there is no compelling evidence for the second stratum in the stem lineage of Theria; (2) gene conversion, which may have occurred between *SMCX/Y* and *UBE1X/Y* in the eutherian lineage, potentially accounts for their apparently lower degree of overall divergence.

## Introduction

Sex chromosomes are widely considered to have differentiated from a pair of autosomes [1], [2]. The differentiation of sex chromosomes has resulted from the suppression of recombination between proto-sex chromosomes [2]–[6]. One likely cause of this suppression is chromosomal inversion [2], [3], [7]. In fact, chromosomal inversion has been frequently observed in the early stage of sex chromosomal differentiation, as reported in plants and animals [8], [9]. However, the genetic linkage required among related sex-determination genes also favors the suppression of recombination [3], [10].

Members of the class Mammalia, including Eutheria (eutherians or placental mammals), Metatheria (marsupials), and Monotremata (monotremes: the most ancient divergence of mammalian taxa), have either a single pair or multiple pairs of X and Y chromosomes [11], [12]. In the common ancestor of Theria (eutherians and marsupials), the X and Y chromosomes evolved from a pair of autosomes syntenic to chromosome 6 in the platypus [13], [14]. However, the sex chromosomes in monotremes originated independently from those in Theria [14]. In eutherians, the sex-determining region Y (*SRY*) gene probably differentiated from its original allele at *SOX3* on the proto-sex chromosome [13], [15]. Although *SRY* has also been identified in several orders of Australidelphia (Australian marsupials) [16], it is absent in monotremes; in platypus, *SOX3* is located on chromosome 6 [13].

Lahn and Page [7] proposed that the process of sequence differentiation between homologous X-Y gene pairs (gametologs) in humans involved 4 successive events of recombination arrest, although it was later found that another recombination arrest occurred near the current pseudoautosomal boundary [17]. Each recombination arrest generated a single segment on the X chromosome that was called an “evolutionary stratum.” Originally Lahn and Page noted that 19 gametologs categorized into 4 groups or strata, based on significant difference of synonymous nucleotide divergence between gametologs (*K_S_* values): 0.94 to 1.25 (stratum 1), 0.52 to 0.58 (stratum 2), 0.23 to 0.36 (stratum 3), and 0.05 to 0.12 (stratum 4) [7]. These strata are ordered by the extent of *K_S_* values from the tip of the long arm to the distal part of the short arm [7], [18]. The *K_S_* values of a stratum depends on the time of recombination arrest. It was proposed that both strata 1 and 2 differentiated in the stem lineage of Theria after the divergence of monotremes. Strata 3 and 4 were formed before the eutherian radiation and after the divergence of prosimian and simian primates, respectively [7], [10], [18]. However, to date, the evolutionary stratum hypothesis proposed by Lahn and Page [7] is not entirely consistent with that proposed by other research groups [19]–[21].

For instance, a pair of *SMCX/Y* genes was recognized as belonging to stratum 2 by Lahn and Page [7] but was placed in stratum 3 by Pearks Wilkerson et al. [19] and in stratum 1 by Sandstedt and Tucker [21]. This inconsistency was caused by the difference of methods and data sets in calculating nucleotide divergences. Lahn and Page, and Sandstedt and Tucker made a simple assumption: they estimated corrected *K_S_* values by the Jukes-Cantor method [7], [21], while Pearks Wilkerson et al. used a Bayesian approach to estimate the divergence time of gametologs based on maximum likelihood trees [19]. In addition, data sets were slightly different among these studies, but marsupial sequences were not included in any cases; Lahn and Page used human and squirrel monkey sequences, Sandstedt and Tucker used mouse sequences, and Pearks Wilkerson et al. used several eutherian sequences [7], [21], [19]. In particular, Pearks Wilkerson et al. [19] reported that the divergence time of eutherian *SMCX/Y* was as short as that of other stratum 3 genes [19]. They also showed that the divergence time of another stratum 2 gene (*UBE1X/Y*) was similar to that of stratum 3 genes. On the basis of the above results, although marsupial gametlogs were not used in the analysis, they suggested that the two genes differentiated independently in the eutherian and marsupial ancestors, but not in the therian ancestor [19]. However, Sandstedt and Tucker [21] reported that nucleotide divergence of mouse *SMCX/Y* is significantly different from that of human *SMCX/Y*, and as large as that of stratum 1 genes, suggesting that *SMCX/Y* differentiated in the therian ancestor.

Previous studies [7], [18] have predicted that the marsupial X chromosome might also contain 2 distinct strata 1 and 2, although this prediction has not yet been tested. Murtagh et al. [22] recently published partial sequences of the wallaby Y chromosome, including 5 novel Y gametologs, and performed phylogenetic analyses on them. However, the presence of marsupial stratum 2 has not been demonstrated. In this study, on the basis of the nucleotide sequences available for marsupial orthologs of several human genes in strata 1 and 2, we showed how sex chromosomal differentiation occurred in the early stages of therian evolution.

## Results

### Divergence of marsupial gametologs

**Figure 1:**
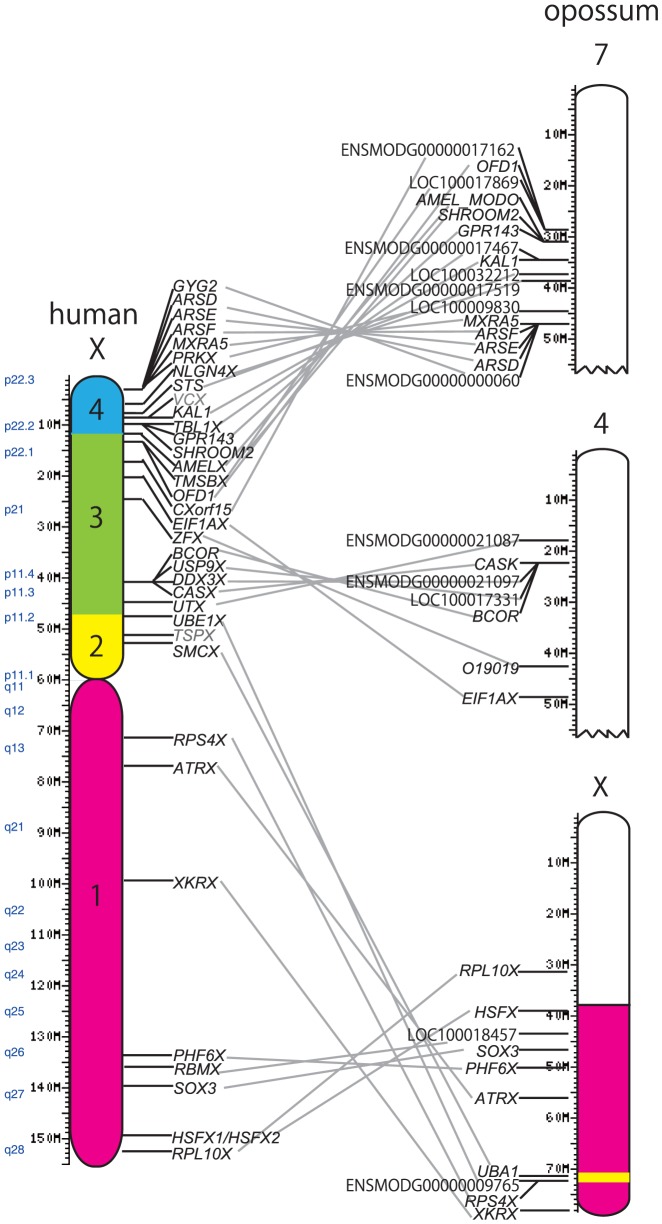
The syntenic relationship between the human X chromosome and the opossum 7, 14, and X chromosomes. Orthologous human and opossum genes are connected by gray lines. In the human X chromosome, each stratum is indicated by a different color (stratum 1, magenta; stratum 2, yellow; stratum 3, green; and stratum 4, blue) [7]. Opossum chromosomal regions homologous with strata 1 and 2 on the human X chromosome are indicated in magenta and yellow, respectively.

We collected 32 available pairs of gametologs in the human or primate genome (Table S1) [7], [17], [18], [23], [24]. Out of the 32 X-linked genes, 5 are located in stratum 1, 3 in stratum 2, 11 in stratum 3, and 13 in stratum 4. Of these, we found 30 orthologs in the opossum genome: 7 are located on the X chromosome, 7 on chromosome 4, and 16 on chromosome 7 (Fig. 1). However, 2 genes, *TSPX* (stratum 2) and *VCX* (stratum 4), were not found in the opossum or other marsupial genome. The 23 genes on the opossum chromosome 4 and 7 are all orthologous to genes in strata 3 and 4 of the human genome (Fig. 1).

Of the 7 genes on the opossum X chromosome (Table 1), 5 are orthologs of human stratum 1 genes (*SOX3*, *RBMX*, *RPS4X*, *HSFX*, and *XKRX*) and 2 are orthologs of stratum 2 genes (*SMCX* and *UBE1X*). Six of the 7 genes have Y gametologs in opossums or other marsupials, while no Y counterpart of *XKRX* was identified. All the 7 genes are located in the long arm of the opossum X chromosome, but the gene order differs from that on the human X chromosome (Fig. 1). In particular, opossum *UBE1X* and *SMCX* are located between *SOX3* and *RPS4X* on the distal end of the long arm, whereas the human orthologs are located at the proximal end of the short arm (Fig. 1). The differences in the ordering of genes between human and opossum X chromosomes may stem from large genomic rearrangements, such as inversion or transposition, in either eutherians or marsupials [25], [26].

**Table 1:** The extent of nucleotide differences (divergences) per synonymous site values of seven gametologs in eutherians and marsupials.

	Eutherians						Marsupials		
	human*1	mouse	cat	dog	cow	previous stratum*3	opossum	Others*4	New stratum
SMCX/Y	0.41±0.018*2 (0.59±0.022)	0.55±0.021 (0.98±0.029)	0.40±0.018 (0.57±0.021)	0.44±0.019 (0.67±0.023)	No Y sequence	2	0.70±0.024 (2.05±0.042)	No sequences	1
SMCX/Ya (5′side)	0.52±0.039 (0.88±0.050)	0.62±0.042 (1.30±0.061)	0.45±0.036 (0.69±0.044)	0.50±0.038 (0.81±0.048)	No Y sequence	1	0.72±0.048 (2.37±0.087)	No sequences	1
SMCX/Yb (3′ side)	0.37±0.020 (0.50±0.024)	0.52±0.025 (0.89±0.032)	0.38±0.021 (0.52±0.024)	0.42±0.022 (0.62±0.026)	No Y sequence	2	0.69±0.028 (1.95±0.047)	No sequences	1
UBE1X/Y	No Y sequence	0.48±0.024 (0.77±0.030)	0.40±0.022 (0.57±0.026)	No Y sequence	0.40±0.056 (0.57±0.067)	2	No Y sequence	0.65±0.029 (1.05±0.089)	1
UBE1X/Ya (5′ side)	No Y sequence	0.49±0.047 (0.79±0.060)	0.41±0.043 (0.60±0.052)	No Y sequence	0.40±0.077 (0.58±0.092)	2	No sequences	No sequences	–
UBE1X/Yb (3′ side)	No Y sequence	0.48±0.028 (0.61±0.031)	0.39±0.025 (0.55±0.030)	No Y sequence	0.40±0.083 (0.57±0.099)	2	No Y sequence	0.65±0.029 (1.05±0.089)	–
RPS4X/Y	0.55±0.052 (0.98±0.070)	No Y sequence	0.64±0.073 (1.42±0.11)	No Y sequence	No Y sequence	1	0.55±0.052 (0.99±0.070)	No sequences	1
XKRX/Y	0.64±0.088 (1.47±0.13)	No Y sequence	No Y sequence	No Y sequence	No Y sequence	1	No Y sequence	No sequences	–
RBMX/Y	0.50±0.040 (0.82±0.051)	0.56±0.062 (1.01±0.084)	No sequences	No Y sequence	0.50±0.045 (0.82±0.058)	1	No Y sequence	0.42±0.037 (0.61±0.044)	1
SOX3/SRY	0.63±0.063 (1.39±0.093)	0.53±0.050 (0.93±0.066)	No X sequence	0.59±0.061 (1.14±0.085)	No X sequence	1	No Y sequence	0.68±0.067 (1.81±0.11)	1
HSFX/Y	0.72±0.076 (2.32±0.14)	No sequences	0.73±0.060 (2.80±0.12)	No Y sequence	0.73±0.052 (2.75±0.10)	1	No Y sequence	0.53±0.077 (0.92±0.10)	1

*1 : In humans, the following genes possess multiple copies (number of copies); *HSFX* (2), *HSFY* (2), *RBMY* (7), *XKRY* (8), and *RPS4Y* (2). For genes with multiple copies, the average value was taken over all X-Y pairs.

*2 : Differences were calculated using a modified version of the Nei-Gojobori method. Values in parentheses were *K_S_* estimated with corrections by the Jukes-Cantor Method. The standard error (values after “±”) was calculated from the maximum variance [53] and by [54].

*3 : Previous stratum was defined by previous studies [7], [18].

*4 : For other sequences, nucleotide differences and divergences in marsupial *UBE1X/Y* and *UBE1X/Yb* are calculated from a comparison of opossum X with kangaroo Y. However, those in marsupial *UBE1X/Ya* could not be calculated because the corresponding nucleotide sequence of kangaroo is not available. Values for *RSMBX/Y*, *SOX3/SRY*, and *HSFX/Y* are gametologs in wallaby, those in dunnart, and opossum X and kangaroo Y, respectively.

Table 1 shows the estimated *p_S_* values in the comparison of conspecific gametologous pairs of genes of marsupials and eutherians. The values were estimated from interspecific gametologs for *UBE1X/Y* and *HSFX/Y*, because of the limited availability of marsupial sequences; however, their *p_S_* values were comparable with those of other gametologs.

Each *p_S_* of *SOX3/SRY*, *RBMX/Y*, *RPS4X/Y*, and *HSFX/Y* in marsupials does not differ significantly from the value for eutherian stratum 1 (Z test). However, the *p_S_* and *K_S_* values of marsupial *SMCX/Y* and *UBE1X/Y* are significantly greater than those of the eutherians (Z test, Z>4.8, P<0.001); yet, they are similar to those of stratum 1 genes (Table 1). Therefore, it appears that *SMCX/Y* and *UBE1X/Y* began to differentiate at the same time as eutherian stratum 1 genes in marsupials.

### Phylogenetic analyses of gametologs in Theria

**Figure 2:**
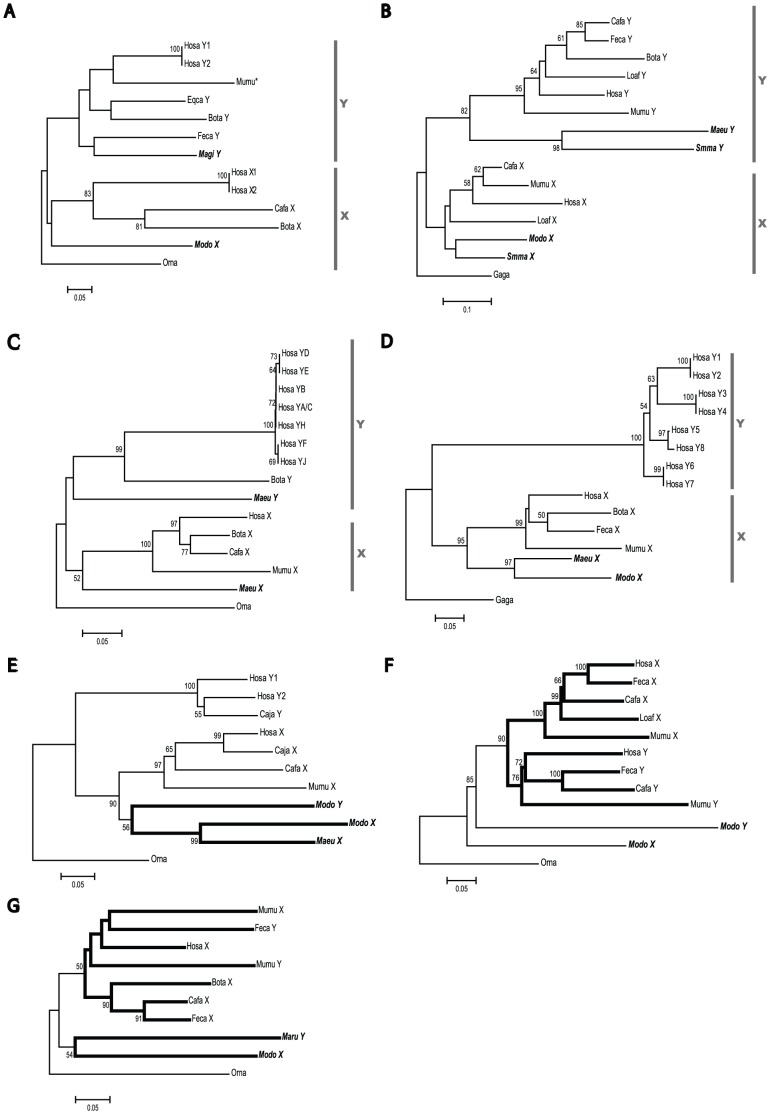
The phylogenetic relationships of 7 gametologs. Neighbor-joining trees were constructed on the basis of the number of synonymous differences per site (*p*_S_). The bootstrap value supporting each internal branch is indicated at the node. Only a bootstrap value of more than 50% is shown. Sequences used for tree construction are listed in Table S1. The number of synonymous sites compared (excluding gaps) and that of operation taxonomy units (OTUs) are as follows: (*A*) *HSFX/Y* (96 sites; 13 OTUs), (*B*) *SOX3/SRY* (70 sites; 15 OTUs), (*C*) *RBMX/Y* (289 sites; 15 OTUs), (*D*) *XKRX/Y* (114 sites; 15 OTUs), (*E*) *RPS4X/Y* (289 sites; 11 OTUs), (*F*) *SMCX/Y* (1280 sites; 12 OTUs), and (*G*) *UBE1X/Y* (147 sites; 10 OTUs). Platypus sequences were used as an outgroup, except in trees *B* and *D*. For trees *B* and *D*, chicken sequences were used as an outgroup. A vertical gray bar beside each tree shows a monophyletic cluster of X- or Y-linked genes. Bold branches in *E*, *F*, and *G* show either marsupial- or eutherian-specific clusters. OTU names in bold indicate marsupials. The abbreviation for species names are as follows: Bota (*Bos taraus*), Cafa (*Canis familiaris*), Caja (*Callithrix jacchus*), Eqca (*Equus caballus*), Feca (*Felis catus*), Gaga (*Gallus gallus*), Hosa (*Homo sapiens*), Loaf (*Loxodonta africana*), Maeu (*Macropus eugenii*), Magi (*Macropus giganteus*), Maru (*Macropus rufus*), Modo (*Monodelphis domestica*), Mumu (*Mus musculus*), Orna (*Ornithorhynchus anatinus*), and Smma (*Sminthopsis macroura*). Mumu* in *HSFX/Y* tree (*A*) is located on chromosome 1 (see Discussion). BotaY sequence was not included in the *UBE1X/Y* tree (*G*) because it is truncated (Fig. S1).

Using synonymous substitutions of the 7 gametologs, phylogenetic analyses were performed to assess whether the differentiation of each of these genes occurred before or after the divergence of therians. The neighbor joining (NJ) trees of *HSFX/Y*, *SOX3/SRY*, *RBMX/Y*, and *XKRX/Y* show that the X- and Y-linked genes are separated into different clusters, each of which include both marsupials and eutherians (Fig. 2*A–D*). This observation is consistent with the prediction that these gametologs differentiated before the divergence of therians. However, this pattern in the NJ trees differs to that of the remaining *SMCX/Y*, *UBE1X/Y*, and *RPS4X/Y* genes (Figs. 2*E–G*).

The NJ trees of *RPS4X/Y*, *SMCX/Y*, and *UBE1X/Y* show that both eutherians and marsupials are not monophyletic with respect to X- or Y-linked genes (Fig. 2*E–G*). Marsupial X- and Y-linked genes form a separate cluster from those of eutherian orthologs, although the bootstrap values supporting this separation are relatively low. This phylogenetic incongruence might be caused by the relatively small number of synonymous sites used in the analysis (Fig. 2); however, a similar pattern was obtained by using the total nucleotide or amino acid sequences in each of the NJ, maximum likelihood (ML), and maximum parsimony (MP) trees (data not shown). Thus, we suggest the possibility that the evolutionary mode of *RPS4X/Y*, *SMCX/Y*, and *UBE1X/Y* was different from that of the other 4 genes.

### Gene conversion between gametologs

**Figure 3:**
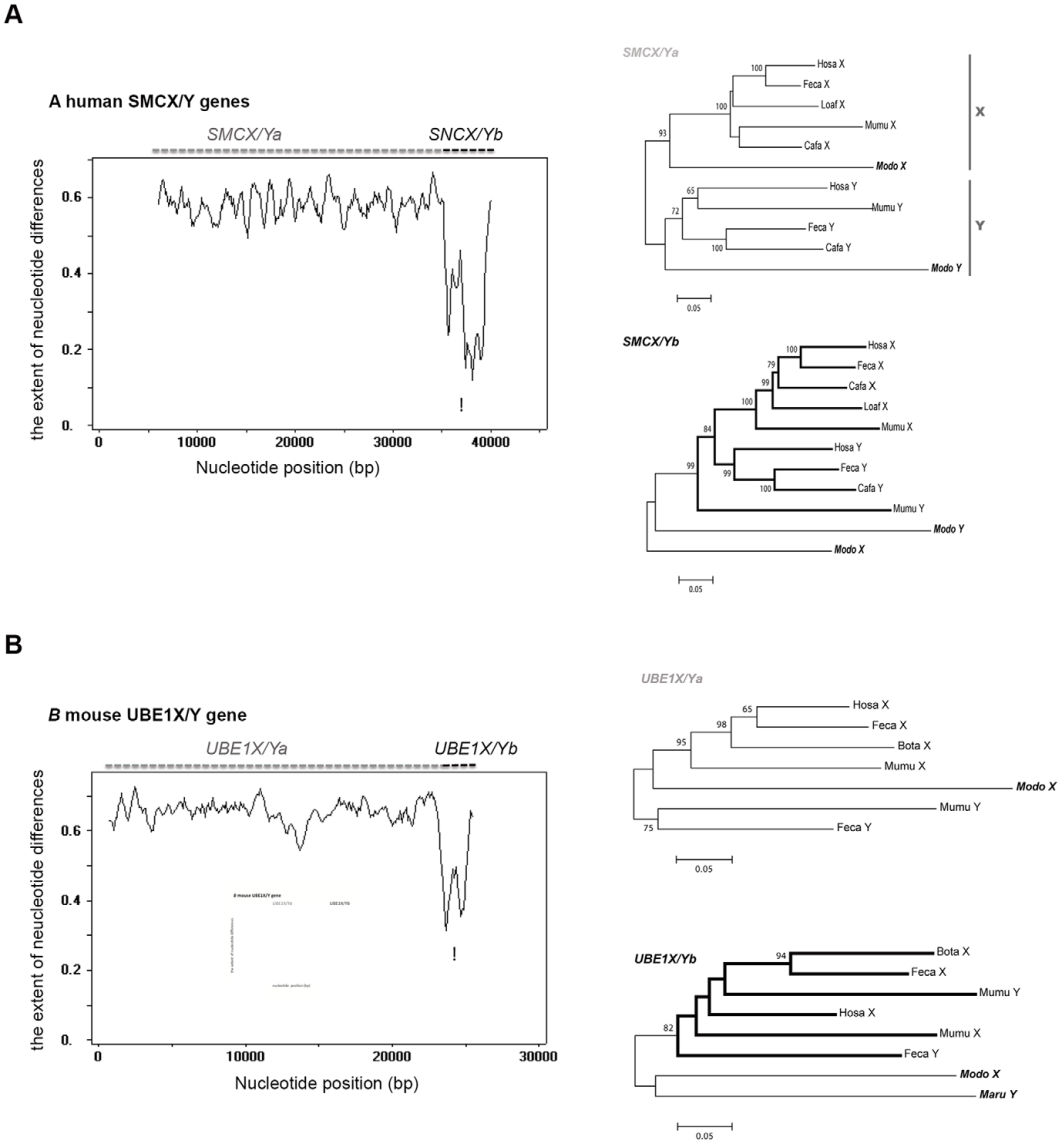
Window analysis of nucleotide divergence and phylogenic relationship of human *SMCX/Y* (A) and mouse *UBE1X/Y* (B) genes. The entire genomic sequences of genes were compared in a window analysis. The window size was 500 bp, with no overlap between adjacent windows. The ordinate represents the extent of nucleotide differences and the abscissa represents position (bp). Position 1 corresponds to the beginning of exon 1 of the X-linked gene. The asterisks indicate the areas showing a statistically significant reduction in nucleotide divergence (*SMCX/Y*: ∼5 kb, *UBE1X/Y*: ∼2 kb). The unrooted tree was based on the number of synonymous differences per site. A bootstrap value of more than 50% is indicated at each node. A vertical gray bar shows a monophyletic cluster of X- or Y-linked genes. Bold branches in B show a eutherian cluster of both X- and Y-linked genes. OTU names in bold are marsupials. The abbreviations for species names are the same as those in Fig. 2. (A) The tree of the 5′ region of the gene (*SMCX/Ya*; exons 1–10) is shown in the left panel and that of the 3′ region (*SMCX/Yb*; exons 11-end) is shown in the right panel. The number of synonymous sites compared was 404 bp (*SMCX/Ya*) or 972 bp (*SMCX/Yb*) without gaps, and 11 OTUs were used. (B) The tree of the 5′ region of the gene (*UBE1X/Ya*; 1–1000 bp) is shown in the left panel and that of the 3′ region (*UBE1X/Yb*; 1001–3180 bp) is shown in the right panel. The number of synonymous sites compared was 332 bp (*UBE1X/Ya*) or 151 bp (*UBE1X/Yb*) without gaps, and 7 OTUs or 8 OTUs were used. In *UBE1X/Ya*, *Maru*Y could not be included because of missing data.

To explore the possibility suggested in the previous section, we examined phylogenetically informative sites at the second codon positions, where nucleotide substitutions are unlikely to be saturated (see File S1 and Fig. S2). In *SMCX/Y*, there are 59 informative sites in total, and importantly the sites indicate two different clustering patterns in phylogeny (Table S2). The phylogenetic relationship supported by the sites is different between the 5′ end of the gene (denoted by *SMCX/Ya*, including the first to the tenth exon) and the 3′ end (denoted by *SMCX/Yb*, including the 11th to the last exon). *SMCX/Ya* supports that the differentiation of X and Y occurred before therian divergence, whereas *SMCX/Yb* indicates that the differentiation occurred after therian divergence (Table S2). Indeed, *p_S_* for eutherian *SMCX/Yb* is significantly (Z test, P<0.001) lower than that of *SMCX/Ya* (Table 1). Furthermore, sliding window analysis of the number of nucleotide differences per site (*p*-distance) also shows large variation across the human *SMCX/Y* gene pair (∼0.6; Fig. 3*A*): *p*-distances of the 3′ end with *SMCX/Yb* are significantly lower than those of *SMCX/Ya* (0.2–0.4 in a ∼5-kb region; Z test, P<0.001; Fig. 3*A*). The phylogenetic analysis for *SMCX/Ya* and *b* consistently supports this region-dependent divergence pattern (Fig. 3*A*). A possible cause for the close relatedness between eutherian *SMCX* and *SMCY* in the b region is an ectopic gene conversion event. Gene conversion was statistically supported by a Runs test and GENCONV software (P<0.001). The topologies of two phylogenetic trees (Fig. 2*F* and 3*A*) indicate the direction of gene conversion is Y to X.

Likewise, we examined mouse *UBE1X/Y*. The reason for using the mouse sequence is that genomic *UBE1X/Y* sequences are available only for this species. Significant gene conversion could not be identified using the Runs test or GENECONV, and actually the *p*-distances of the 5′ side and 3′ side of CDS are not different from each other (Table 1). However, sliding window analysis of the gene including intronic sequences showed the low extent of nucleotide differences in mouse *UBE1X/Y* (∼0.4 in a ∼2-kb region; Z test, P<0.001) at the 3′ region (Fig. 3*A and B*), compared to 5′ region. Besides, in the 3′ region, the phylogeny shows the monophyletic relationship of eutherian *UBE1X/Y*, indicating possible gene conversion in eutherians (Fig. 2 and 3*B*). In contrast, the 5′ region exhibits separate clustering of X and Y gametologs, although the marsupial Y sequence is unavailable in this region (Fig. 3*B*).

The large *p_S_* value of *RPS4X/Y* indicates that X- and Y-linked genes separated before therian divergence (Table 1). Nevertheless, the phylogeny based on the number of nucleotide substitutions shows that therian *RPS4X* or *Y* genes are not respectively monophyletic, and that the single marsupial cluster of *RPS4X* or *Y* genes is more closely related to the eutherian *RPS4X* gene cluster than to the eutherian *RPS4Y* gene cluster (Fig. 2*E*). The phylogenetic tree based on the number of amino acid differences supports the same topology as that based on the number of nucleotide differences. However, it turns out that the branch length leading to *RPS4Y* is significantly shorter in opossums than in humans. For instance, the opossum *RPS4Y* branch (2.75±0.10) is approximately one-sixth of the human *RPS4Y* branch (16.25±0.25) (Fig. S3). The short branch leading to opossum *RPS4Y* indicates that ectopic gene conversion also occurred from *RPS4X* to *RPS4Y* in the marsupial.

### Dating of sex chromosomal differentiation in Theria

**Figure 4:**
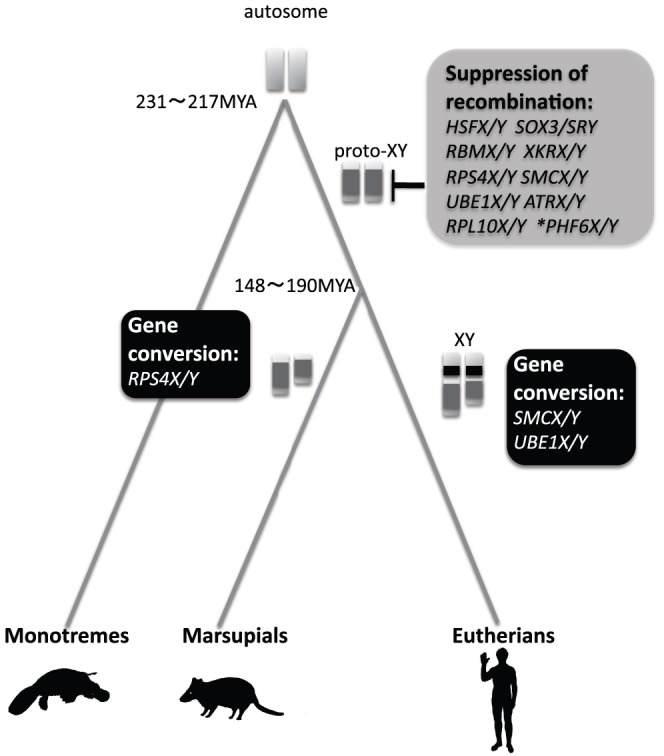
Schematic diagram of sex chromosome evolution in Theria. After the divergence of Theria from monotremes, recombination was suppressed in at least 10 genes on the proto-XY chromosome in the therian ancestor; this is indicated by gray color on the chromosome. In the stem lineage of marsupials and eutherians, a gene conversion occurred in *RPS4X/Y* or both *SMCX/Y* and *UBE1X/Y*, respectively; this is indicated by back color on the chromosome. An asterisk means that it is not clear whether *PHF6X/Y* diverged at the same time as the differentiation of the other gametologs (see text).

At least 7 gametologs (*HSFX/Y*, *SOX3/SRY*, *RBMX/Y*, *XKRX/Y*, *RPS4X/Y*, *SMCX/Y*, and *UBE1X/Y*) might have differentiated simultaneously in the stem lineage of Theria (Fig. 4). Except for *RPS4X/Y*, *SMCX/Y*, *UBE1X/Y*, which experienced possible gene conversion, the *K_S_* value is 1.33±0.63 across the gametologs in Theria. From this value, the divergence time of these gametologs was inferred as 224–173 million years ago (MYA; see Materials and Methods), if the synonymous substitution rate was 5.95×10^−9^ to 7.67×10^−9^ per site per year. Even if we assume a different substitution rate in a different lineage, the estimated divergence time does not differ much from that calculated here (data not shown). Therefore, we suggest that therian sex chromosomal differentiation occurred around or after the divergence of Theria from monotremes (i.e., about 231–217 MYA) [27].

## Discussion

### Loss of gametologs in therian evolution

Gene losses were often observed on sex chromosomes [19], [24], [28], [29], [30]. In this study, we also observed that not all the 7 pairs of gametologs were found in all the species used (Table 1) and six of them were consistent with the previous results. Previous studies showed that *UBE1Y* has been lost at least twice in primates: once in the Catarrhini (hominoids and Old World monkeys) ancestor and once in the marmoset lineage [28]. Similarly, *RPS4Y* has been lost at least 3 times in eutherian lineages, leading to rodents, artiodactyls (pigs and cows), and horses [19], [29], [30]. While *XKRY* is only present in primates, the large *K_S_* value of *XKRX/Y* does not support its emergence in primates [24]. Rather, it is more likely that *XKRY* was lost in non-primates. In addition to these reported genes, we found that *HSFX/Y* are also absent in mice and rats; however, an *HSFY-*like processed gene is present on an autosome (Fig. 2*A*; Mumu*). It is likely that in rodents, *HSFY* retrotransposed to an autosome, after which both *HSFX/Y* were lost.

Both *TSPX*/*Y* genes are also absent in marsupials. It is more likely that *TSPX*/*Y* genes emerged in eutherians, rather than having been lost in marsupials, because a BLAST search could not identify any *TSPX*-like gene in vertebrates, except eutherians. However, the divergence time between *TSPX/Y* genes estimated using *K_S_* value (*K_S_* = 1.06±0.20; *p_S_* = 0.56±0.05) is 178–138 MYA. Therefore, we cannot rule out the possibility that both *TSPX/Y* genes arose before therian divergence (190–148 MYA); however, they were lost in marsupials.

### Gene conversion between gametologs

Despite large sequence divergences between therian gametologs, our results showed the possibility of gametologous gene conversion in eutherian *SMCX/Y* and *UBE1X/Y*. Human *SMCX/Ya* (5′-end of the gene) showed a *p_S_* value of 0.52±0.039, while *SMCX/Yb* (3′-end of the gene) showed a *p_S_* value of 0.37±0.020 (Table 1). Therefore, the *p_S_* of the entire gene was averaged as 0.41±0.018, which is lower than the average *p_S_* of stratum 1 genes (0.61±0.085; Z test, P<0.001). The *p*_S_ values observed in the mouse, cat, and dog *SMCX/Ya* and *b* are all similar to those in humans (Table 1), indicating that gene conversion at *SMCX/Yb* occurred in the eutherian ancestor. This observation is consistent with the previous result of Sandstedt and Tucker [21], who reported that the nucleotide difference in mouse *SMCX/Y* is high in almost the entire region, except the 3′-end [21].

To some extent, a local reduction in nucleotide differences between *UBE1X/Y* gametologs should decrease the nucleotide difference in the entire *UBE1X/Y*, as in the case of *SMCX/Y* (Table 1 and Fig. 3*B*). Excluding such a local region, the *p_S_* value becomes similar to that of the other 5 genes in stratum 1 (Table 1 and Fig. 3*A and B*).

Pearks Wilkerson et al. [19] estimated shorter divergence times of eutherian stratum 2 genes (*SMCX/Y* and *UBE1X/Y*) than that calculated by Lahn and Page [7], indicating that these 2 genes diverged independently in the eutherians and marsupials. However, the authors did not consider the possibility of gene conversion, and therefore, they probably underestimated the divergence time of stratum 2. In any event, if ectopic gene conversion events occurred in eutherian *SMCX/Y* and *UBE1X/Y*, the presence of stratum 2 proposed by Lahn and Page [7] is not substantiated. Our conclusion is that recombination arrest between the proto-sex chromosomes in the therian ancestor formed only a single stratum.

### Marsupial sex chromosomal differentiation

The human X-linked gametologs are ordered from the small to the large value of *p_S_*; however, this is not the case in the opossum. In addition to the 6 gametologs in marsupials (Table 1), another gene named *ATRX* could also be examined. Both marsupial and eutherian *ATRX* are located on the long arm of the X chromosome (Fig. 1), and only *ATRY* is found in marsupials [31], [32]. The extent of marsupial *ATRX/Y* divergence is large (*p*_S_ = 0.54±0.020; *K*_S_ = 0.94±0.027), as observed for the other 6 pairs of marsupial gametologs (Table 1 and Fig. S4 *A*; P>0.05). The *ATRX/Y* phylogeny shows that this pair of gametologs diverged in the therian ancestor, although the monophyletic relationship of *ATRX* is only weakly supported (Fig. S4). In the 5 novel Y gametologs recently reported by Murtagh et al. [22], 3 gametologs (i.e., *MECP2X/Y*, *HCFC1X/Y*, and *HUWE1X/Y*) differentiated in the marsupials after its divergence from the eutherians. This information is based on the monophyletic relationships of the marsupial gametologs. However, 2 other genes (i.e., *RPL10X/Y* and *PHF6X/Y*) showed the possibility of differentiation before the therian divergence. While this interpretation is not entirely reliable, our re-analysis also supported their conclusion (Fig. S4 *B* and *C*). The extent of wallaby *RPL10X/Y* divergence (*p*_S_ = 0.72±0.066; *K*_S_ = 1.31±0.089) is as large as that of the other 6 marsupial gametologs. Moreover, the *p*_S_ value is greater than that of orthologous wallaby and human *RPL10X* (*p*_S_ = 0.58±0.059; *K*_S_ = 1.06±0.080). This variation indicates that *RPL10X/Y* possibly differentiated before the divergence of marsupials and eutherians. In contrast, the extent of *PHF6X/Y* divergence (*p*_S_ = 0.37±0.074; *K_S_* = 0.52±0.087) is smaller than that in other marsupial gametologs. This rather small value is similar to that of orthologous genes between the human and wallaby (*p*_S_ = 0.39±0.075; *K*_S_ = 0.56±0.089). The small value between *PHF6X/Y* means that the synonymous nucleotide substitution rate somehow slowed down compared with other genes because the *p*_S_ and *K*_S_ values between the human and wallaby are significantly smaller in *PHF6X* (*p*_S_ = 0.39±0.075; *K*_S_ = 0.56±0.089) than in *RPL10X* (*p*_S_ = 0.58±0.059; *K*_S_ = 1.06±0.080). Yet, it is likely that *PHF6X/Y* diverged before therian divergence, although it is not clear whether *PHF6X/Y* diverged at the same time as the other gametologs.

### Conclusions and perspective

We proposed a single recombination arrest in the therian ancestor in the early process of sex chromosomal evolution in mammals and provided evidence for regional gene conversion between eutherian gametologs categorized as belonging to the so-called stratum 2. In the therian ancestor, at least 9 pairs of gametologs probably differentiated simultaneously (Fig. 4; *HSFX/Y*, *SOX3/SRY*, *RBMX/Y*, *XKRX/Y*, *RPS4X/Y*, *SMCX/Y*, *UBE1X/Y*, *ATRX/Y*, and *RPL10X/Y*). Simultaneous differentiation of these gametologous gene pairs may have been facilitated by chromosome-wide recombination suppression between the proto-sex chromosomes. Under this sheltering effect, it appears that functional diversification of X- or Y-linked alleles subsequently took place, becoming responsible for sex determination and sex differentiation.

Although the above conclusion was drawn from a limited amount of data, if the genomic sequence of the entire marsupial Y chromosome is completed, additional gametologs are likely to become available. These might include gametologs that differentiated in the therian ancestor and in the marsupial lineage. Such information would allow us to discuss directly marsupial sex chromosomal evolution, and to identify how many strata marsupials have.

## Materials and Methods

### Sequences used

Nucleotide sequences of genes on the sex chromosomes from the mammals listed below and their homologs from chicken (*Gallus gallus*) (Table S1) were obtained from NCBI (http://www.ncbi.nlm.nih.gov/) and Ensembl databases (release 62; http://uswest.ensembl.org/index.html). The mammals used in this study included a monotreme: platypus (*Ornithorhynchus anatinus*); marsupials: gray short-tailed opossum (*Monodelphis domestica*), tammar wallaby (*Macropus eugenii*), red kangaroo (*Macropus rufus*), eastern gray kangaroo (*Macropus Giganteus*), and stripe-faced dunnart (*Sminthopsis macroura*); and eutherians: human (*Homo sapiens*), marmoset (*Callithrix jacchus*), mouse (*Mus musculus*), dog (*Canis familiaris*), cat (*Felis catus*), cow (*Bos taurus*), horse (*Equus caballus*), and elephant (*Loxodonta africana*).

### Detection of gametologs in mammals

We performed BLASTN searches to identify orthologs of human gametologs against each of the mammalian genome sequences with a cut-off *e* value of 10^−4^. Homologs were identified as sequences that displayed more than 70% similarity to a query sequence. To confirm orthology with genes on the human X chromosome, sequences adjacent to the homologs were examined using a program called “Synteny” in Ensembl. For genes on the Y chromosome, orthologs could not be identified because of frequent genome rearrangements on the Y chromosome in each species. Therefore, homologous sequences identified by a BLASTN search were regarded as orthologs. The gene-name abbreviations follow the standard nomenclature for human genes.

Because the marsupial *HSFY* sequence was unavailable in the databases, the nucleotide sequence was obtained from male eastern gray kangaroos by using the polymerase chain reaction (PCR) method. Genomic DNA was extracted from a liver sample (which was provided by the Kanazawa Zoo of Yokohama City, Japan, in 2000) and the DNA was used as a template in PCR amplification. Genomic DNA (10 ng) was in 20 L of 1× Ex Taq PCR buffer containing 0.2 mM of each deoxyribonucleotide triphosphate (dNTP), 0.5 mM of each of the 2 primers, and 1 unit of TaKaRa Ex Taq DNA polymerase (TaKaRa). A set of primers (5′-TGATTGAAGAAAATGCTTTTCAGGCTTT-3′ and 5′-GCCTCTTTTAAAATTAGGATT-3′) was designed on the basis of evolutionary conserved sequences in the platypus ortholog and eutherian *HSFY*. The following PCR procedure was used: 95°C for 30 s, followed by 35 amplification cycles of denaturation for 15 s at 95°C, annealing at 58°C for 60 s, and extension at 72°C for 60 s. A final extension was performed for 10 min at 72°C. The PCR fragment of ∼700 bp was directly sequenced using an Applied Biosystems 3130 genetic analyzer in both directions. The obtained sequence was deposited into DDBJ (accession number: AB667854).

### Phylogenetic and molecular evolutionary analyses

The obtained sequences were translated into amino acids and were then aligned using ClustalX software [33], with manual corrections (Fig. S1). In this study, both nucleotide and amino acid sequences were used in the following analyses. Nucleotide divergence was calculated using the corrected number (*K_S_*) and uncorrected number (*p_S_*) of synonymous nucleotide differences per synonymous site, according to a modified version of the Nei–Gojobori method (assuming transition/transversion bias (R) = 1), with the MEGA 5.03 program [34]. Multiple hit corrections for *K_S_* were performed using the Jukes–Cantor model [35]. For nucleotide and amino acid sequences, phylogenetic trees were constructed using the 3 different methods available in the MEGA 5.03 program [34]: NJ [36], ML [37], and MP [38]. For nucleotide sequences, NJ trees were reconstructed on the basis of *p_S_* values, ML trees were constructed using the Kimura 2-parameter model [39], and MP trees were constructed using default conditions. For amino acid sequences, ML and MP trees were also constructed. Default conditions were used for MP, and the substitution model for ML was the Jones–Taylor–Thornton model [40]. Bootstrap resampling with 1000 replications assessed the reliability of these trees.

### Detection of gene conversion

We investigated whether phylogenetically informative sites were distributed uniformly in the alignment of gametolog sequences by using 2 statistical tests. The first test was the two-sample runs test [41], which used the informative sites in the alignment of 4 operation taxonomy units for both eutherian and marsupial gametologs. We tested whether the run of informative sites was significantly clustered. The second test was the global test in the GENECONV program (version 1.81) [42] under the default conditions. The global test for reciprocal recombination or gene conversion events was performed with 10,000 permutations of the sequence alignment to assess significance. In addition, we examined the heterogeneity of nucleotide divergence along the sequences by using a window analysis (window size = 500 bp, no overlaps) implemented in DnaSP v5 [43].

### Estimation of nucleotide substitution rate

Due to male-biased mutations in the germ line [44], nucleotide sequences at silent sites (i.e., synonymous sites or sites in a non-coding region) on the X and Y chromosomes evolve at different rates. The X chromosome was present in the female germ line during two-thirds of the evolutionary time and in the male germ line during the remaining one-third. Assuming a 1∶1 sex ratio, the mutation rate of X chromosomal genes (m_X_) is the sum of two-thirds of the female mutation rate (m_f_) and one-third of the male mutation rate (m_m_), whereas the mutation rate of Y chromosomal genes (m_Y_) is the same as m_m_. In several eutherians, m_m_ is 2 to 6 times as high as m_f_ (α = m_m_/m_f_ = 2–6) [45]–[49]. For a conservative estimate, we assumed that α = 2. We also estimated the autosomal nucleotide substitution rate per site per year (m_A_ = 1/2(m_m_+m_f_)) from a comparison of genomic sequences of opossum–human orthologs. The average *K_S_* between the 2 species was 1.02 (0.76–1.44) [50]. This was translated into m_A_ = 2.68×10^−9^–3.45×10^−9^, assuming that the divergence time between eutherians and marsupials is 148–190 MYA [27], [51], [52]. We estimated m_X_ = 2/3 m_f_+1/3 m_m_ = 2.38×10^−9^–3.07×10^−9^ and m_Y_ = m_m_ = 3.57×10^−9^–4.60×10^−9^, respectively, yielding m_XY_ = 5.95×10^−9^–7.67×10^−9^ for the XY gametolog comparison.

## Supporting Information

Figure S1 **The alignment of amino acid sequences of gametologs.** The alignment used in Figures 2A–G, 3A–B, S3 and S4 is shown in (A–I). (A) *HSFX/Y* (96 sites; 13 OTUs), (B) *SOX3/SRY* (70 sites; 15 OTUs), (C) *RBMX/Y* (289 sites; 15 OTUs), (D) *XKRX/Y* (114 sites; 15 OTUs), (E) *RPS4X/Y* (152 sites; 11 OTUs), (F) *SMCX/Y* (*SMCX/Yab*: 1280 sites; 12 OTUs), (G) *UBE1X/Ya* (329 sites; 7 OTUs), (H) *UBE1X/Y* (*UBE1X/Yb*: 147 sites; 10 OTUs), and (I) *ATRX/Y* (862 sites: 9 OTUs). In (G), the sequences of ModoY and Orna are missing and could not be aligned.
(PDF)

Figure S2 **The topology among 4 eutherian and marsupial X/Y genes.** If gametologs differentiated before speciation, the phylogeny of X- or Y-linked genes would be monophyletic (*A*). If gametologs differentiated after speciation or if a lineage-specific gene conversion occurred between X and Y genes, the phylogeny of each species would be monophyletic (*B*). The third case, showing the different topology of (A) and (B), might potentially occur (*C*). The abbreviations in this figure are as follows: EX (a eutherian X gene); EY (a eutherian Y gene); MX (a marsupial X gene); and MY (a marsupial Y gene).
(EPS)

Figure S3 **The phylogenic relationship of** ***RPS4X/Y*** **.** The unrooted tree was based on the number of amino acid substitutions (No. of differences). The bootstrap value was 100% at each node. The number of sites that were compared was 263 amino acids without gaps, with 4 OTUs being used. Branches in bold show *RPS4X* and *Y* in the opossum. An asterisk indicates that the branch length leading to *RPS4Y* was significantly shorter in marsupials than in humans. OTU names in bold are marsupials. The abbreviations for species names are the same as those in Fig. 2.
(EPS)

Figure S4 **The phylogenic relationship of marsupial gametologs.** The neighbor joining (NJ) tree was based on the number of synonymous differences per site (*p*_S_). A bootstrap value of more than 50% is indicated at each node. (A) In *ATRX/Y*, the number of synonymous sites compared was 1473 bp without gaps, with 9 OTUs being used. (B) In *RPL10X/Y*, the number of synonymous sites compared was 213 bp without gaps, with 9 OTUs being used. This NJ tree did not form 1 cluster of eutherian and marsupial X-linked genes. ML and MP trees of nucleotides (644 bp) showed monophyletic relationships of the eutherian and marsupial X-linked genes, although the bootstrap value was low. (C) In *PHF6X/Y*, the number of synonymous sites compared was 95 bp without gaps, with 10 OTUs being used. In trees of (B) and (C), the alignments were applied to the supplementary information of Murtagh et al [22]. The vertical gray bar beside the tree indicates a monophyletic cluster of X-linked genes. OTU names in bold are marsupials. The abbreviations for species names are the same as those in Fig. 2.
(EPS)

Table S1 **Accession number of nucleotide sequences used in this study.**
(XLSX)

Table S2 **The phylogenetic informative sites at the second position of the codon.** The number of sites to support each topology of fig. S2 was shown. A, B or C means topology A, B or C in fig. 4. *UBE1X/Yb* is the region with the low divergence and *UBE1X/Ya* is the rest.
(TIF)

File S1 **The distribution and number of phylogenetically informative sites.** We examined the distribution and number of phylogenetically informative sites by using only the second positions of codons, in which substitutions were unlikely to be saturated. This analysis excluded *XKRX/Y*, for which the Y homolog was not present in the opossum genome. For simplicity, 4 OTUs were used: the X and Y sequences from the opossum (marsupial X and Y: MX and MY) and a eutherian (human or cat), denoted by EX and EY (eutherian Y). Each phylogenetically informative site supports one of 3 possible topologies (Fig. S1). One topology (topology A: Fig. S1A) is supported by the partition as ([EX, MX], [EY, MY]), in which the inner parentheses indicate nucleotides that are shared. Gametologs that differentiated before therian divergence show the partition ([EX, MX], [EY, MY]), whereas differentiation after divergence produces the topology ([EX, EY], [MX, MY]). Furthermore, while the partition of ([EX, MY], [MX, EY]) is not consistent with early therian divergence, it could occur by chance (topology C: Supp Fig. S1 *C*). Table S2 shows the number of phylogenetically informative sites for each topology of the 4 genes (there are too few informative sites in *HSFX/Y* and *RPS4X/Y* to determine a topological category). The informative sites in *SOX3*/*SRY* and *RBMX/Y* support the differentiation of gametologs before therian divergence (Table S2), which is consistent with the topology of their nucleotide trees. *SMCX/Y* and *UBE1X/Y* support topology B (Fig. S2 *B*), suggesting differentiation after therian divergence (Table S2), which is not consistent with the topology of the nucleotide trees.
(DOCX)
