# Correction: No Evidence for a Second Evolutionary Stratum during the Early Evolution of Mammalian Sex Chromosomes

**DOI:** 10.1371/journal.pone.0097169

**Published:** 2014-05-09

**Authors:** The PLOS ONE Staff

Figure 3 incorrectly contains characters and text that should not be present. Please see the correct version of Figure 3 below.

**Figure 3:**
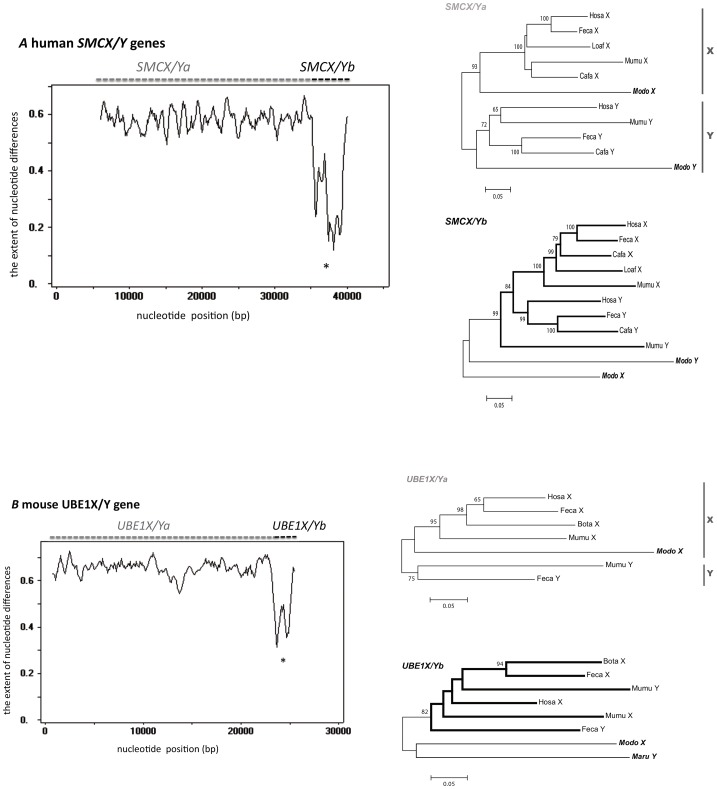
Window analysis of nucleotide divergence and phylogenic relationship of human *SMCX/Y* (A) and mouse *UBE1X/Y* (B) genes. The entire genomic sequences of genes were compared in a window analysis. The window size was 500 bp, with no overlap between adjacent windows. The ordinate represents the extent of nucleotide differences and the abscissa represents position (bp). Position 1 corresponds to the beginning of exon 1 of the X-linked gene. The asterisks indicate the areas showing a statistically significant reduction in nucleotide divergence (*SMCX/Y*: ∼5 kb, *UBE1X/Y*: ∼2 kb). The unrooted tree was based on the number of synonymous differences per site. A bootstrap value of more than 50% is indicated at each node. A vertical gray bar shows a monophyletic cluster of X- or Y-linked genes. Bold branches in B show a eutherian cluster of both X- and Y-linked genes. OTU names in bold are marsupials. The abbreviations for species names are the same as those in Fig. 2. (A) The tree of the 5′ region of the gene (*SMCX/Ya*; exons 1–10) is shown in the left panel and that of the 3′ region (*SMCX/Yb*; exons 11-end) is shown in the right panel. The number of synonymous sites compared was 404 bp (*SMCX/Ya*) or 972 bp (*SMCX/Yb*) without gaps, and 11 OTUs were used. (B) The tree of the 5′ region of the gene (*UBE1X/Ya*; 1–1000 bp) is shown in the left panel and that of the 3′ region (*UBE1X/Yb*; 1001–3180 bp) is shown in the right panel. The number of synonymous sites compared was 332 bp (*UBE1X/Ya*) or 151 bp (*UBE1X/Yb*) without gaps, and 7 OTUs or 8 OTUs were used. In *UBE1X/Ya*, *Maru*Y could not be included because of missing data.
