# Supplementary material for: No Evidence for a Second Evolutionary Stratum during the Early Evolution of Mammalian Sex Chromosomes
Source: PLoS One. 2012 Oct 19;7(10):e45488. doi: 10.1371/journal.pone.0045488 (PMC3477149; doi:10.1371/journal.pone.0045488)
Supplement: Figure S1 — The alignment of amino acid sequences of gametologs. The alignment used in Figures 2A–G, 3A–B, S3 and S4 is shown in (A–I). (A) HSFX/Y (96 sites; 13 OTUs), (B) SOX3/SRY (70 sites; 15 OTUs), (C) RBMX/Y (289 sites; 15 OTUs), (D) XKRX/Y (114 sites; 15 OTUs), (E) RPS4X/Y (152 sites; 11 OTUs), (F) SMCX/Y (SMCX/Yab: 1280 sites; 12 OTUs), (G) UBE1X/Ya (329 sites; 7 OTUs), (H) UBE1X/Y (UBE1X/Yb: 147 sites; 10 OTUs), and (I) ATRX/Y (862 sites: 9 OTUs). In (G), the sequences of ModoY and Orna are missing and could not be aligned. (PDF) [file pone.0045488.s001.pdf]

Figure S1

A HSFX/Y

```
#Hosa_X1 EASFRRPHPD VPGEDNLLSL PFPQKLWRLV SSNQFSSIWW DDSACRVINQ KLFEKEILKR DVAHKVFATT SIKSFFRQLN LYGFRKRRQL VSILNK
#Hosa_X2 .......... .......... .......... .......... .......... .......... .......... .......... .......... ......
#Cafa_X  .PLLK.LRT. ALAGS...C. ...K.....L N.SR.T.... E.RKSIGLKE ...Q....EW .RPD...E.D CT...I.... ...LS.LCKA .RV...
#Bota_X  RRLQDNLP.N PKK.EDI.G. S..R....I. EDAA.T.AC. N.ENMV..EE D..RM...Q. RGMDQI.E.D .....ISE.. ..E...IHPG C.AGKM
#Modo_X  KR.YFPLTNE SVE.NEFF.. T..R...KI. E.DR.K.V.. NEDS.II.DE .Q.RE...EN KGPCRI.E.D CM...I.... ....S.L.HE ALP.S.
#Hosa_Y1 GSLLES.SYV SEKD.DF... N..R...KI. E.D..K..S. .ENT.I...E E..K....ET KAPYRI.Q.D A....V.... ....S.IQ.E S.V.S.
#Hosa_Y2 GSLLES.SYV SEKD.DF... N..R...KI. E.D..K..S. .ENT.I...E E..K....ET KAPYRI.Q.D A....V.... ....S.IQ.E S.V.S.
#Feca_Y  GSLMK..RYV SEKDHDF... T..R...NI. E.D..K.... .ENTSI..DE E..K..V.E. KAPFRI.E.G .M..LV...H ....S.M..E ..V.S.
#Eqca_Y  GSLIK...HV SDEDNDF... T..R...KIT E..H.K.... .ENTSI...E E..KQ.V.EK KAPFRI.G.D NM...V.... ......M.PT ..V.I.
#Mumu_Y  RFLIK..PHA SEEDS..F.M T..R...KI. G.DK.K.... .EDTYI...E E..K..V.E. KAPFRI.E.D .M..LV.... ......M..G I.ASC.
#Bota_Y  DLVIK..CYV SEEVT.F... T......NI. E.D..E.... .ERT.I..HE E..K..V.E. KAPFRI.E.K .M..LI.... ....S.K..N I.L.S.
#Magi_Y  RPLIK..RF. .GE.NDF... T..K...KI. E.E..K.... .SDT.V..DE E..K..V.E. KGPFRI.E.D CM...I.... ....S.M..E ..V.S.
#Orna    GPLVKKRRY. GSDSNDFFAF T..K...KII E.D..Q.... NNDNSI..DE E..K..V.ES KGPFRI.E.D CM...I.... ....S.I.YA ..AM..
```

B SOX3/SRY

```
#Hosa_Y VKRPMNFIVW SRDQRRKMAL ENPRMRNSEI SKQLGYQWKM LTEAEKWPFF QEAQKLQAMH RKYPNYKYRP
#Cafa_Y .R.....L.. .......... ...Q.Q.... .......... .......... E...R..... ....D.....
#Feca_Y .......M.. .......V.. ...QTQ.... .......... ..Q....... E...R...L. ....G.R...
#Bota_Y .......... ..ER...V.. ...K.K..D. ......E..R ..D...R... E...R.L.I. ....G.....
#Mumu_Y .......M.. ..GE.H.L.Q Q..S.Q.T.. .....CR..S ......R... ....R.KIL. ........Q.
#Loaf_Y .......L.. FC........ ...Q...... ..L....... ..D...L..L E...R.R... ..........
#Smmc_Y .......M.. .QT....V.. Q..K.H.... .....VT..L .SDS..R..I D..KR.RDK. KQVSD...Q.
#Maeu_Y .......MI. ..S....V.. ...K.H.... ..H..FT... .PDN..Q..I D..ER.R.K. .EF.D...Q.
#Hosa_X .......M.. ..G....... ...K.H.... ..R..AD..L ..D...R..I D..KR.R.V. ME..D.....
#Mumu_X .......M.. ..G....... ...K.H.... ..R..AD..L ..D...R..I D..KR.R.V. ME..D.....
#Cafa_X .......M.. ..G....... ...K.H.... ..R..AD..L ..D...R..I D..KR.R.V. ME..D.....
#Loaf_X .......M.. ..G....... ...K.H.... ..R..AD..L ..D...R..I D..KR.R.V. ME..D.....
#Smmc_X .......M.. ..G....... ...K.H.... ..R..AD..L ..D...R..I D..KR.R.V. ME..D.....
#Modo_X .......M.. ..G....... ...K.H.... ..R..AD..L ..D...R..I D..KR.R.V. ME..D.....
#Gaga   .......M.. ..G......Q ...K.H.... ..R..AD..L .SD...R..I D..KR.R.V. ME..D.....
```

C RBMX/Y

```
#Hosa_YA/C NRETNEKMLK AVFGKHGPIS EVLLIKDRTS KSRGFAFITF ENPADAKNAA KDMNGKSLHG KAIKVEQAKK PSFQSGRRRP PASSRNRSPS GSLRRGSRGG
#Hosa_YH   .......... .......... .......... .......... .......... .......... .......... .......... .......... ..........
#Hosa_YB   .......... .......... .......... .......... .......... .......... .......... .......... .......... ..........
#Hosa_YF   .......... .......... .......... .......... .......... .....T.... .......... .......... .......... .......S..
#Hosa_YJ   .......... .......... .......... .......... .......... .....T.... .......... .......... .......... .......S..
#Hosa_YD   .......... .......... .......... .......... .......... .......... .......... .......... .......... ..........
#Hosa_YE   .......... .......... .......... .......... .......... .......... .......... .......... .......... ..........
#Bota_Y    SA..T.ES.E .E...Y.H.V .........N .......... .S.....D.. .E....F.D. .T......N. ...E...QKL QPPA...GHP RN....GS..
#Maeu_Y    .VG....D.E S....Y.H.V K...M..Q.N .......V.. .S..A..D.. R.....A.D. .S......N. ...E....G. .PAP.S.G.P RG....GS.E
#Hosa_X    .T.....A.E .....Y.R.V ....M....N .......V.. .S.....D.. R.......D. ........T. ...E....G. .PPP.S.G.P RG....GS..
#Maeu_X    .T.....A.E .....Y.R.V ....M..... .......... .S.....D.. R.....L.D. .S......T. .T.E....G. .PPP.S.G.P RG....GS..
#Bota_X    .T.....A.E .....Y.R.V ....M....N .......V.. .S.....D.. R.......D. ........T. ...E....G. .PPP.S.G.P RG....GS..
#Mumu_X    .T.....A.E .....Y.R.V ....M....N .......V.. .S.....D.. R.......D. ........T. ...E....GL .PPP.S.G.P RG....GS..
#Cafa_X    .T.....A.E .....Y.R.V ....M....N .......V.. .S.....D.. R.......D. ........T. ...E....G. .PPP.S.G.P RG....GS..
#Orna      .T.....A.E .....Y.R.V ....M....N .......V.. .S.....D.. R.......D. ........T. ...E....G. .PPP.S.G.P RG....GS..

#Hosa_YA/C RGWLPHEGHL DDGGYTPDKS YSRGLIPVKR GPSSRSGGPP PKKSAPSAVA RSNSWMGSQG PMSRRENYGV PPRRATISSW RNDRMSTRHD GYATNDGHSN
#Hosa_YH   .......... .......... .......... .......... .......... .......... .......... .......... .......... ..........
#Hosa_YB   .....Q.... .......... .......... .......... .......... .......... .......... .......... .......... ..........
#Hosa_YF   .......... .......... .......... .......... .......... .......... .......... .......... .......... ..........
#Hosa_YJ   .......... .......... .......... .......... .......... .......... .......... .......... .......... ..........
#Hosa_YD   .....Q.... .......... .......... .......... .......... .......... .......... .......... .......... ..........
#Hosa_YE   .....Q.... .......... .......... .......... .......... .......... .......... .......... .......... ..........
#Bota_Y    ARGRM.G.NF G.N..SLNT. S...PY...K .....CQ..S ..R....VQG ..S.G.RGR. .VL.....RG G...QPV..R .DNYV.P.DY ....K.SY.G
#Maeu_Y    ARGP.RG..M ..S..SLNNG S.M.PLL... .APPQ..Q.S ..R....GPV Y.SRQ..GR. QL.G.DS..G ....ESL..R .DVH..P.D. S.S.KESY.S
#Hosa_X    TRGP.RG..M .....SMNN. S...PL.... ..PP...... ..R....GPV ..S.G..GRA .V.G.DS..G ....EPLP.R .DVYL.P.D. ..S.K.SY.S
#Maeu_X    TRGP.RG..M ..S..SLNNG S...PL.... ..PP...... ..R....GPV ..S.G..GRA .V.G.D...G ....DPMP.R .DVY..P.D. ..N.K.SY.S
#Bota_X    TRGP..G..M .....SMNN. S...PL.... ..PP...... ..R....GPV ..S.G..GRA .V.G.DS..G ....EPLP.R .DVYL.P.D. ..S.K.SY.S
#Mumu_X    TRGP.RG..M .....SMNT. S...PL.... ..PP...... ..R....GPV ..S.GL.GRA .V.G.DG..G ....EPLP.R .DVYL.P.D. ..S.K.SY.S
#Cafa_X    TRGP.RG..M .....SMNN. S...PL.... ..PP...... ..R....GPV ..S.G..GRA .V.G.DS..G ....EPLP.R .DVYL.P.D. ..S.K.SY.S
#Orna      ARGP.RG..M ..S..SLNNG S...PL.... ..PP...... ..R....GPV ..S.G..GRA .V.G.D...G ....EPMP.R .DVY..P.D. ..S.K.SY.S

#Hosa_YA/C RDHSSRGYRY APPSRGHAYR DYGHSSSWDE HSSRGYSYHD GYGLGRDHSE HLSGSSYRDA LQRYGTSHGA PPARGPRSYG GSTCHAYSR
#Hosa_YH   .......... .......... .......... .......... .......... .......... .......... .......... .........
#Hosa_YB   .......... .......... .......... .......... .......... .......... .......... .......... .........
#Hosa_YF   .......... .......... .......... .......... .......... .......... .......... .......... .........
#Hosa_YJ   .......... .......... .......... .......... .......... .......... .......... .......... .........
#Hosa_YD   .......... .......... .......... .......... .......... .......... .......... .......... .........
#Hosa_YE   .......... .......... .......... .......... .......... .......... ......A... .......... .........
#Bota_Y    ..P...DTK. .....DY..H .........D .....H.D.. ...RD..Y.. .R..G....S YRS..G.... VSG...P... RRHYDD...
#Maeu_Y    ..A..WDV.. ..LP.EYV.. .......R.. YT.....DR. S..RD..Y.D .Q..GR...S YES..Y.RR. ..P...SC.. ..RYED...
#Hosa_X    ..P...DT.. ...P.DYT.. .......R.D YP.....DR. ...RD..Y.D .P..G....S YES..N.RS. ..T...P... ..RYDD...
#Maeu_X    ..Q...DN.. ...P.DY... .......R.. YQ.....DR. ...RD..Y.D .P..G....S YES..N.RS. ......P... ..RYDD...
#Bota_X    ..P...DT.. ...P.DYT.. .......R.D YP.....DR. ...RD..Y.D .P..G....S YES..N.RS. ..T...P... ..RYDD...
#Mumu_X    .EP...DT.. ...P.DYT.. .......R.D YP.....DR. ...RD..Y.D .P..G....S YES..N.RS. ..T...P... ..RYDD...
#Cafa_X    ..P...DT.. ...P.DYT.. .......R.D YP.....DR. ...RD..Y.D .P..G....S YES..N.RS. ..T...P... ..RYDD...
#Orna      ..P...DT.. ...P.DY... .......R.. YP.....DR. ...RD..Y.D .P..G....S YES..N.RS. ......P... ..RYDD...
```

D XKRX/Y

```
#Hosa_X  VPLRVVLMVF SLVSVTYGAT LCNMLAIQIK YDDYKIRLGP LEVLITIWRT LEITSRLLIL VLFSATLKLK AVPFLVLNFL IILFEPWIKF WRSGAQMPNN
#Mumu_X  ....A...A. ..I....... .......... .......... ......V... .......V.. .......... .......... .......V.. ..........
#Bota_X  .......I.. ..I....... .......... ..E....... .......... .......V.. .......... .....L.... .......V.. ..........
#Feca_X  ....A..... ..I....... .......... ..E....... .......... .......V.. .......... .....L.D.. .......V.. ..........
#Maeu_X  M....I.II. A.I....... .......... .....VHIHM T.IIV....S .......I.. ...V....T. .L..FMI..I .......VR. .K........
#Modo_X  M.I.....A. A.I....... .......... .....FH.HI CDIIV....S .......... ...V....SR .L...II.YV .......VR. ..........
#Gaga    ..V.AI..GI C........L V..V....V. .....VQ.R. .AF..VL..S ...ST.VAV. ....TVF.HW II.IALA.L. VVF.Q..VQ. ....TRL.D.
#Hosa_Y1 MFINSIADDI FPLISCV..I H..I...RTG N.FAA.K.QV IKLILM..HS .V.I.PVVT. AF.P.S..QG SLH..LIIYF VL.LT..LE. SK..THL.S.
#Hosa_Y2 MFINSIADDI FPLISCV..I H..I...RTG N.FAA.K.QV IKLILM..HS .V.I.PVVT. AF.P.S..QG SLH..LIIYF VL.LT..LE. SK..THL.S.
#Hosa_Y3 IFTNSIADYM FPLISCV..I HR.I...HTC NNFAA.K.WV IKFIVM..HS .VTI..VVT. AF.P.S...R TLH..LIIYF VLFLAS.PEI .KT.VHFHS.
#Hosa_Y4 IFTNSIADYM FPLISCV..I HR.I...HTC NNFAA.K.WV IKFIVM..HS .VTI..VVT. AF.P.S...R TLH..LIIYF VLFLAS.PEI .KT.VHFHS.
#Hosa_Y5 LFISSIADDM F.LTTCVA.I H..T...HTS N.FPD.K.QA IKFIVM.RCS .V.I..VVT. EF.P.S..QR SL.L.LIIYF VL.LA..LE. .K...HLH..
#Hosa_Y6 MFINSIADDI FPFISCV..I H..I...HTS N.FAA.K.QV IKFIVVVL.S .VVI.CVMA. AFLP.S...R SL...LIMYF VL.LA..LE. .K...HP.S.
#Hosa_Y7 MFINSIADDI FPFISCV..I H..I...HTS N.FAA.K.QV IKFIVVVL.S .VVI.CVMA. AFLP.S...R SL...LIMYF VL.LA..LE. .K...HP.S.
#Hosa_Y8 LFISSIADDM F.LTTCVA.I H..T...HTS N.FPD.K.QA IKFIVM.RCS .V.I..VVT. EF.P.S..QR SL.L.LIIYF VL.LA..LE. .K...HLH..

#Hosa_X  IEKNFSRVGL VVLI
#Mumu_X  .......... ....
#Bota_X  .......... ....
#Feca_X  .......... ....
#Maeu_X  .......... ....
#Modo_X  ......Y... ...A
#Gaga    .........V ...F
#Hosa_Y1 TKN.S.M..Y GC.S
#Hosa_Y2 TKN.S.M..Y GC.S
#Hosa_Y3 T.N.S.MA.Y GC.S
#Hosa_Y4 T.N.S.MA.Y GC.S
#Hosa_Y5 T.N.S.M..Y GC.P
#Hosa_Y6 T.N.S.M..Y GC.F
#Hosa_Y7 T.N.S.M..Y GC.F
#Hosa_Y8 T.N.S.M..Y GC.P
```

E RPS4X/Y

```
#Hosa_X  ARGPKKHLKR VAAPKHWMLD KLTGVFAPRP STGPHKLREC LPLIIFLRNR LKYALTGDEV KKICMQRFIK IDGKVRTDIT YPAGFMDVIS IDKTGENFRL
#Caja_X  .......... .......... .......... .......... .......... .......... .......... .......... .......... ..........
#Mumu_X  .......... .......... .......... .......... .......... .......... .......... .......... .......... ..........
#Cafa_X  .......... .......... .......... T......... .......... .......... .......... .......... .......... ..........
#Modo_X  .......... .......... .......... .......... .......... .......... .......... ........T. ..V....... .E....H...
#Maeu_X  .......... .......... .......... ......M... ..I......H .......... .......... V....Q..P. ..V....... .E....R...
#Hosa_Y1 .......... .......... .......... .......... ....V..... .......... .......... ......V.V. .......... .E....H...
#Hosa_Y2 .......... .......... .......... .......... ....V..... .......... ......H.L. ......V... .....I.... .E....H...
#Caja_Y  ........R. LV........ .......... .....R.... ....V..... .......... .........R ......V... ..V.L..... .E....H...
#Modo_Y  .......... .......... .......... .......... .......... .......... .......... .......... .......... .E....H...
#Orna    .......... .......... .......... .......... .......... .......... .......... .......... .......... .E....H...

#Hosa_X  IYDTKGRFAV HRITPEEAKY KLCKVRKIFV GTKGIPHLVT HDARTIRYPD PL
#Caja_X  .......... .......... .......... .......... .......... ..
#Mumu_X  .......... .......... .......... .......... .......... ..
#Cafa_X  .......... .......... .......... .......... .......... ..
#Modo_X  V......... ....A..... .......T.. ...A...... .......... ..
#Maeu_X  V......... ....A..... .......T.. ...A...... ......C... ..
#Hosa_Y1 V......... ....V..... ........T. .V........ .......... .V
#Hosa_Y2 V.N...C... ....V..... ........T. .......... .......... ..
#Caja_Y  V......... ...RV...E. ........TM .......... .......... .V
#Modo_Y  V......... ....A..... .......... A......... .......... ..
#Orna    V......... ....A..... .......... .......... .E........ ..
```

F SMCX/Y （SMCX/Yab)

```
#Hosa_X DFLPPPECPV FEPSWAEFRD PLGYIAKIRP IAEKSGICKI RPPADWQPPF AVEVDNRFTP RIQLNELEAQ TRVKLNYLDQ IAKFWEIQGS SLKIPNVERR
#Mumu_X .......... .......... .......... .......... .......... .......... .......... .......... .......... ..........
#Cafa_X .......... .......... .......... .......... .......... .......... .......... .......... .......... ..........
#Feca_X .......... .......... .......... .......... .......... .......... .......... .......... .......... ..........
#Loaf_X .......... .....EFP.T R.A..GEKIR PIASP..... .......... .....TSVY. ....I..D.. .......... .......... ..........
#Modo_X ..V....... .......... .......... .......... ...P...... .......... .......... .......... .......... ....S.....
#Hosa_Y E......... ........Q. .......... .......... .......... .......... .V........ .......... .......... .........K
#Mumu_Y .......... .......... .......... .......... .......... .......... .......... .......... .......... .........K
#Cafa_Y .......... ...T...... ..D..T.... .......... .......... .......... .......... .......... .......... ..........
#Feca_Y .......... ...T...... .......... .......... .......... ....E..... .......... .......... .......... ..........
#Modo_Y E......... .....E..A. .FAF.H.... ...QT....V ...PA..... .CD..KH... .......... ......F... ......L..C T....H...K
#Orna   MRR.RHSLDS LQVTEVGG.R GQDEKPASQN SNSDQARERA EEGE...... .......... .......... .......... .......... ..........

#Hosa_X ILDLYSLSRL NYPPGKNIGS LLRSHYERIV YPYEMYQSGA NLVQCNTRPF DNEEKDKEYK PHSIPLRQSV QPSKFNSYGR RAKRLQPDPE PTEEDIEKNP
#Mumu_X .......... .......... .......... .......... .......... .......... .......... .......... .......... ..........
#Cafa_X .......... .......... .......... .......... .......... .......... .......... .......... .......... ..........
#Feca_X .......... .......... .......... .......... .......... .......... .......... .......... .......... ..........
#Loaf_X .......... .......... .........I .......... .......... .......... .......... .......... .......... ..........
#Modo_X ........KI GWA.HRVTKD SCEHVG.LTL SLPGPFPLLL S.Q....... .S........ .......... .......... .......... ..........
#Hosa_Y .......... H......... .........I .....F.... .H.....H.. ...V...... .......... .....S..S. .......... ........H.
#Mumu_Y .......N.. ...S...... .........I ....IF.... .......D.. .S..R..... .......... .....SC.S. .G.....E.. ..........
#Cafa_Y .......... ...A...... .........I .....F.... .......Y.. .......... ..G....... ...T.S..S. .......... ..........
#Feca_Y .......... .......... .........I .....F.... .......H.. .......... ..G....... .....S..S. .......... ..........
#Modo_Y ....FQ.NKM GFA...AV.. HI.A.....L ...NLF.... S.LCLQKPNL TTDT...... ..D..Q.... ..VETCPPA. ....MRAEAT NIKTEPGEA.
#Orna   .......... .......... .......... .......... .....TA... .S........ .......... .......... .......... ..........

#Hosa_X ELKKLQIYGA GPKMMGLGLM AKDKTLRKKP TVVVKEESGG DVTMRLRNHS FIESYVCRMC SRGDEDDKLL LCDGCDDNYH IFCLLPPLPE IPKGVWRCPK
#Mumu_X .......... .......... .......... .......L.. .......... .......... .......... .......... .......... ..........
#Cafa_X .......... .......... .......... .......L.. .......... .......... .......... .......... .......... ..........
#Feca_X .......... .......... .......... .......... .......... .......... .......... .......... .......... ..........
#Loaf_X .......... .......... .......... .......P.. .......... .....I.... .......... .......... .......... ..........
#Modo_X .......... .......... ....S..... .......V.P EP.......N .......... A......... .......... .......... ....I.....
#Hosa_Y .........P .......... ....DKTVH. ..T..D.QS. GG..Q..... ..D..I.QV. .......... F......... .......... ..R.I.....
#Mumu_Y .......... ....I....K ..E......A .I...G.ASE FG..Q..... .MN.....I. .....V..F. .....S.... ........S. V.........
#Cafa_Y .......... .......... .........R A.LM...PRE .E..Q..T.. ..DL....I. .......... .......T.. ....I..... ..R.......
#Feca_Y .......... .......... .......... ..LM...PRE .G.....S.N .VGT....I. .....V.... .....G.... ....M..... ..........
#Modo_Y .VRTHNLRRR MGCPVPKCEN E.EIRGTI.D ..EK..HV.E IPKS.SKSTN AVDL...LL. GS.NDE.R.. .......S.. T...I...HD V...D.....
#Orna   .......... .......... .......... .........V .L........ ..D..I.... G......... .......... .......... ..........
                                                                                                          a←:→b
#Hosa_X CVMAECKRPP EAFGFEQATR EYTLQSFGEM ADSFKADYFN MPVHMVPTEL VEKEFWRLVN SIEEDVTVEY GADIHSKEFG GFPVSDNKRH LTPEEEEYAT
#Mumu_X .......... .......... .......... .......... .......... .......... .......... .......... ......S... ..........
#Cafa_X .......... .......... .......... .......... .......... .......... .......... .......... ......S... ..........
#Feca_X .......... .......... .......... .......... .......... .......... .......... .......... ......S... ..........
#Loaf_X .......... .......... .......... .......... .......... .......... .......... .......... ......S... ..........
#Modo_X .......... .......... .......... .......... .......... .......... .......... .......... ...I..ST.. .SS......A
#Hosa_Y .IL....Q.. .........Q ..S....... .....S.... .......... .........S .......... .......... .....NS.QN .S...K....
#Mumu_Y .IL....S.. .........Q .......... .......... .........V .........S .......... .......... ....NNS.WD .S...K...A
#Cafa_Y .I........ .........Q .......... .........S .......... .........S .......... .......... .....SSQ.I .S........
#Feca_Y .I........ .........Q .......... .......... .........H .........S .......... .......... .....SS.KN .S........
#Modo_Y .LAQ..NK.Q ........A. D...RT.... ..A..S.... .......... .........S T......... ....A..... ....R.G.IK .S.G....LD
#Orna   .......... .......... .......... .......... .......... .......... .......... .......... ...I..G..Q .S.......A

#Hosa_X SGWNLNVMPV LEQSVLCHIN ADISGMKVPW LYVGMVFSAF CWHIEDHWSY SINYLHWGEP KTWYGVPSLA AEHLEEVMKK LTPELFDSQP DLLHQLVTLM
#Mumu_X .......... .......... .......... .......... .......... .......... .......... .......... .......... ..........
#Cafa_X .......... .......... .......... .......... .......... .......... .......... .......... .......... ..........
#Feca_X .......... .......... .......... .......... .......... .......... .......... .......... .......... ..........
#Loaf_X .......... .......... .......... .......... .......... .......... .......... .......... .......... ..........
#Modo_X .......... .......... .......... .......... .......... .......... ........F. .......... .......... ..........
#Hosa_Y .......... .D........ .......... .......... .......... .......... .......... .........M .......... ..........
#Mumu_Y C......... .D........ .......... .......... .......... .......... .......... .....D...R .......... ..........
#Cafa_Y .......... .D........ .......... .......... .......... .......... .......... ..Q......R .......... ..........
#Feca_Y .......... .D........ .......... .......... .......... .......... .......... ..Q......R .......... ..........
#Modo_Y ......N... M.....A..T ...C...L.. .....C..S. .......... .......... .....A.GY. ..Q..D.... .A....V... ........I.
#Orna   .......... .......... .......... .......... .......... .......... ........F. .....D.... ......E... ..........
```

```
#Hosa_X NPNTLMSHGV PVVRTNQCAG EFVITFPRAY HSGFNQGYNF AEAVNFCTAD WLPAGRQCIE HYRRLRRYCV FSHEELICKM AACPEKLDLN LAAAVHKEMF
#Mumu_X .......... .......... .......... .......... .......... .......... .......... .......... .......... ..........
#Cafa_X .......... .......... .......... .......... .......... .......... .......... .......... .......... ..........
#Feca_X .......... .......... .......... .......... .......... .......... .......... .......... .......... ..........
#Loaf_X .......... .......... .......... .......... .......... .......... .......... .......... .......... ..........
#Modo_X ......A... .......... .......... .......... .......... .......... .......... .......... .......... ..........
#Hosa_Y .......... .......... .......... .......... .......... .......... .......... .......... ..F..T.... ..V.......
#Mumu_Y .......... .......... .......... .......... .......... ...V...... .......... .......... ..F....... ..V.......
#Cafa_Y .......... .......... .......... .......... .......... .......... .......... .......... ..F....... ..V.......
#Feca_Y .........I .......... .......... .......... .......... ...T...... .......... .......... ..F....... ..V.......
#Modo_Y ......A... ..Y....... .......... .......F.. ........V. ...L....V. ...L.N.... ...D.M.... .SKADV..VV V.ST.Q.D.A
#Orna   .......... .......... .......... .......... .......... .......... .......... .......... .......... ..........

#Hosa_X IMVQEERRLR KALLEKGITE AEREAFELLP DDERQCIKCK TTCFLSALAC YDCPDGLVCL SHINDLCKCS SSRQYLRYRY TLDELPAMLH KLKVRAESFD
#Mumu_X .......... .......... .......... .......... .......... .......... .......... .......... .......... ..........
#Cafa_X .......... .......... .......... .......... .......... .......... .......... .......... .......... ..........
#Feca_X .......... .......... .......... .......... .......... .......... .......... .......... .......... ..........
#Loaf_X .......... .......... .......... .......... .......... .......... .......... .......... .......... ..........
#Modo_X .......... .......... .......... ......A... .......... .......... ...D.....P T.K....... .......... ..........
#Hosa_Y .......... .......V.. .......... .......... .......... .......... .......... .......... ......T... ...I......
#Mumu_Y .......... .T........ .......... .......... .......... .....S.... .......... RN........ .........Q ...I......
#Cafa_Y .......... .......... .......... .......... .......... .......... .......... .......... .......... ...I......
#Feca_Y .......... .......... .......... .......... .......... .......... .......... .R........ .......... ..........
#Modo_Y ..IED.KT.. ETVRKL.VID S..MD..... .......... ....M.GVS. SCK.GL.... H.VE...S.P TYKYK.G... ...D.YP.MN A..L....YN
#Orna   .......... .......... .......... ......A... .......... .......... ...E.....P ..K....... .......... ..........

#Hosa_X TWANKVRVAL EVEDGRKRSL EELRALESEA RRFPNSELLQ RLKNCLSEAE ACVSRALGLV SGQEAGREAL ASLPSSPGLL QSLLERGQQL GVEVPEAQQL
#Mumu_X .......... .......... .......... .......... .......... .......... .......... V.Q....... .......... ..........
#Cafa_X .......... .......... .......... .......... Q......... .......... .......... .......... .......R.. ..........
#Feca_X .......... .......... .......... .......... Q......... .......... .......... .......... ........L. ..........
#Loaf_X .......... .......... .......... .......... .......... .......... ......YGSM RCWGRDSL.S I.I..KCYKR WTVSTGTLEI
#Modo_X ...SQ..A.. .L........ .......... ....H..... Q..D..RR.. .......R.. .SR...QT.. ....A.L.H. P....QAHL. .......G..
#Hosa_Y .......... .........F .......... .......... ........V. ..IAQV.... ...V.R.... .T........ R......... .......H..
#Mumu_Y N.....QA.. .........F .......... .......... ...K..T... ..I.QV...I .NS.DR.... T...Y.LEI. ...M.K.... R......H..
#Cafa_Y .......... .........F .......... .......... ..R..MH... ....QV.... .....R.... ...CP.V..M R....K.... ..D....H..
#Feca_Y N......... .....Q...F .......... .....N.... ..R...N... ....QV...I .....R.... TL..P.V... R......... ..R....H..
#Modo_Y E..LN.NE.. .AKINN.K.. VNFK.....T KK..DND..R H.RLVTQD.D K.A.V.QQ.L N.KRQTQKL. SEEMP.AAE. .E..DVSFEF D..L.QLSEM
#Orna   .......... .L........ .......... ....H.D... ...S..TQ.. K...Q..... ...DS.Q... EA....LAQ. PG......R. .......ER.

#Hosa_X QRQVEQALDE VKRTLAARRG LAVMRGLLVA GASVAPSPAV DKARAEQELL TIAERWEEKH LCLEARQKHP PATLEAIIHE AENIPVHLPN IQALKEALAK
#Mumu_X .......... .....S.... ..I....... .......... ...Q...... .......... .......... .......... .......... ..S.......
#Cafa_X .......... .....S.... .......... .......... ...Q...... .......... .......... ........R. .......... ..........
#Feca_X .......... .....S.... .......... .......... .......... .......... .......... .......... .......... ..........
#Loaf_X S.KLT...EL KR.WPSR..A .....E.V.. APV....LL. ..P....... .......... .......... ........R. .......... ..........
#Modo_X KL..Q..... ...A.P.Q.. .........S ..G.T..... .......... A......... .......... ........R. ...L...... .L.....G.
#Hosa_Y .Q........ ..QA.S.H.. .VI.Q....M ..KI.S..S. .......... .......... F......... ........R. T......... ........T.
#Mumu_Y EELL.....Q ..QA.SGQ.H .VI.KK...M .TK..S..S. N......... ....C..... F..K.S...S .....V..R. ......Y... ..S.....T.
#Cafa_Y .Q.......D ..KA.S.Q.. .VI.Q....T .TKI.S..C. .......... .......... F......... ........R. .......... .....D....
#Feca_Y .Q........ ..QA.SGK.. .VI.Q..S.T ..K..S..S. .......... ...K...... F......... ........R. .......... .....D....
#Modo_Y RIRL....E. .H.ACPSSLT .DD..R.IDL .VGL..YS.. E..M.R.... .VS.H.DD.R SLIK..PR.S LNS.A.AVK. I.E..AY... GL...D.VQ.
#Orna   R..IQ.G.E. ...A.P.Q.. ........TS ..G....... E..L...... .......... I......... ........R. .......... .L........

#Hosa_X ARAWIADVDE IQNGDHYPCL DDLEGLVAVG RDLPVGLEEL RQLELQVLTA HSWREKASKT FLKKNSCYTL LEVLCPCADA GSDSIKRSRW MEKELGLYKD
#Mumu_X .......... .......... .......... .......... .......... .......... .......... .......... ....T..... ..........
#Cafa_X .......... .......... .......... .......... .......... .......... .......... .......... ....T..... ..........
#Feca_X .......... .......... .......... .......... .......... .......... ...R...... .......... ....T..... ..........
#Loaf_X .......... .......... .......... .......... .......... .......... .......... .......... ....T..... ..........
#Modo_X .......... .......... .......... .......D.. .......... .......... .......... .......... ..E.M..D.. R......DR.
#Hosa_Y .Q........ .......... .......... .......... .......... .......... .......... .......... ....T..... ...A....Q.
#Mumu_Y .Q......N. .......... .......... .....E.... ....N..... ...K...... .......... .......... ..V.T..... I..M......
#Cafa_Y .Q........ .......... ....C..... .....S.... .......... ........RM .......... ........H. ....S..R.. I....R..R.
#Feca_Y .Q........ .......... ........I. .......... .......... .......... .......... .......VN. ....S..... IG......R.
#Modo_Y ..D.LQE.EA L.A.GRV.V. ET.ME..SR. .SI..H.NS. PR..SL.AEV QA.K.C.AN. ..ME..P.S. ......RC.V .AVGL..KQR LKEPIPSG.S
#Orna   ........E. .......... .......... .......... .......... .......... .......... .......... ....M..TK. K.........
```

```
#Hosa_X TELLGLSAQD LRDPGSVIVA FKEGEQKEKE GILQLRRTNS AKPSPAATSV CVCGQVPAGV ALQCDLCQDW FHGRCVSVPR LLKFLCPLCM RSRRPRLETI
#Mumu_X .......... .......... .......... .......... ......TA.I .......... .......... ......T... .......... ..........
#Cafa_X .......... .......... .......... .......... ......T..I ......L..A .......... .......... .......... ..........
#Feca_X .......... .......... .......... .......... .......... .......... .......... .......... .......... ..........
#Loaf_X .......... .......... .......... .......... .........I .......... .......... .......... .......... ..........
#Modo_X .......... ........L. .......... ...H...A.A T..R.PDSPS .....A.V.E .......R.. ...C...A.. .S........ ..........
#Hosa_Y .......... .......... .......... .......... ......P..I .......... V......... ...Q.....H .......... ..........
#Mumu_Y .......... ........M. .....E.... ...H..HI.. .....N...I .I....C... S......H.. ...Q..T..H .......... ..........
#Cafa_Y .......... .......... .......... .......... .........I ....K..... T......... ...Q.....H I......... ..........
#Feca_Y ......T... ......L... .......... ...H...... ..SR.S...I .......... T......... ...Q...... .......... ..........
#Modo_Y LSD.ERALTE SKETA.AMAT LG.ARL..M. ALQS..AA.E G.LLSQEIKI .L.QKA..AP MI..E..R.A ..TG..A..S I.VW...Y.Q ..EK.P..K.
#Orna   ..S....... ........L. .......... .........A ...C.PTSAA SCVCGQ.M.E .......R.. ...G..AP.. .G........ ..........

#Hosa_X LALLVALQRL PVRLPEGEAL QCLTERAISW QGRARQALAS EDVTALLGRL AELRQRLQAE PLREGSGKMP KVQLLENGDS VTSPEKVALD LELLSSLLPQ
#Mumu_X .......... .......... .......... ......V... .E........ .......... SGG..T.N.. .......... ........T. ......I...
#Cafa_X .......... .......... .......... .......... .......... .......... .......... .......... ........P. ..........
#Feca_X .......... .......... .......... .......... .......... .......... .......... .......... ........PN ..........
#Loaf_X .......... .......... .......... .......... .......R.. ...H.LV... .MPK...... .......... .......... ..........
#Modo_X ........K. .......... ........G. .....R.... RE.S...... .......HRS .ESRDG..E. QMP.HR...G MKN....SP. .........R
#Hosa_Y .......... .......... ........G. .D...K.... .......RQ. .....Q...K .I.....NIS .......... .....NM.P. ..........
#Mumu_Y .S...G.... S......... ........G. .......... .......KQ. EKS..Q..D. L.T.N...IL .EE.VL.EER IK.S..IVP. K...P...S.
#Cafa_Y .......... .......... ........V. .......... K...T...Q. ....HQ.... T......R.. ..P..P.V.. TS.H..I.S. ..V.....S.
#Feca_Y .......... .......... ........V. .......... ........Q. G....Q..S. .........H ..P..P.V.G ...H....P. ..V......H
#Modo_Y .P..AS...I R......D.. RYMI..TVN. .H..Q.M.S. GSLKLV.DQV GSGLLYSRWQ AAMSF.LPDW DRTY.HSPF. TGRSCIPLHE AQ..QVS..E
#Orna   ........K. .......... ........T. .D....V... R..AG..... D.......GD ..K.S.CNP. ..KAALENGD S.T...ASP. .........R

#Hosa_X LTGPALELPE ATRAPLEELM LEGDLLEVTL DENHSIWQLL QAGQPPDMER RLELEKAERH GSRARGRALE RRRRRKDRGG
#Mumu_X .S..V..... .......... M......... .......... .......LK. Q......... ...T...... ..........
#Cafa_X ....V..... .......... M......... .......... .......L.. .......... .......... ..........
#Feca_X ....V..... .......... .......... .......... .......... .......... .......... ..........
#Loaf_X ....M..... .......... M......... .......... ...K...L.. ........H. .......... K.........
#Modo_X .E..V..... .A.V...... M......... .......... ...R...LP. .I.......Q .A.G.N..P. K.....E.TD
#Hosa_Y ....V..... .I........ M......... .......... .......LD. ......F.HQ ...T.S.... .....Q.Q.R
#Mumu_Y ....V..... .......... M......... ...Y...... ....N.NL.. H.....P.NP .NWSEEQTP. ...Q.RVVLS
#Cafa_Y .S..V.D... ...V...... .......... ....R..... .......L.. C.....P.HK ...T...... K....QVDL.
#Feca_Y ....V.D... .......... .......... .......... .......L.. HM....H.HK .N.T.S.T.. K....Q.L.R
#Modo_Y IQEIYQT.LT KPSPTQHPDR GSPVG.TSEK N.CCRGKRDG IGNLERKLK. ...R.GSSIE LRERIKKTRT PKKKKIKDMN
#Orna   .E..V..... TA.G...... M......... .......... ..ARE..LT. ...PCLILQL PRSVARAFRF GH.GFNMD.S
```

G UBE1X/Y (UBE1X/Ya)

```
#Hosa_X MSSSPLSKKR RVSGPDPKPG SNCSPAQSVL SEVPSVPTNG MAKNSEADID EGLYSRQLYV LGHEAMKRLQ TSSVLVSGLR GLGVEIAKNI ILGGVKAVTL
#Bota_X .......... .......... ......H... .......A.. .....D.... .......... .......... .......... .......... ..........
#Mumu_Y ....V..... K.....SSLD .SW..TY..M FG..PG...E .S..K.M... .S........ .......H.. A....I...Q .......... ..C.......
#Mumu_X .......... .......... ........A. ...S...... .......... .S........ .......M.. .......... .......... ..........
#Feca_Y .......... C.P....ETS TD..SSY..M FNM.LG.S.. .Q.KN.T... .......... ......RH.. .......... .......... ..........
#Feca_X .......... .......... ......H... .......... .......... .......... .......... .......... .......... ..........
#Modo_X .......... .......... ......HP.P .DA.TA.A.. ....N..... ........P. Q.LMPGPL.T LTPTPSNPYW SISGDSGQ.. ..........

#Hosa_X HDQGTAQWAD LSSQFYLREE DIGKNRAEVS QAELNSYVPV TAYTGPLVED FLSGFQVVVL TNTPLEDQLR VGEFCHNRGI KLVVADTRGL FGQLFCDFGE
#Bota_X .......... .......... .......... .......... S......... ...D...... ..S....... ......SH.. .......... ..........
#Mumu_Y ....I..... .....C.... ........I. .......... F......I.E .......... ......Y..Q ......SH.. .......... V.........
#Mumu_X .....T.... .......... .......... .......... .......... ...S...... ..S...A... ......S... .......... ..........
#Feca_Y .......... .......... .......... .......... .T...A.... ..T....... ..A.....Q ......SH.. .......... ..........
#Feca_X .......... .......... .......... .......... S......... .......... .......... ......S... .......... ..........
#Modo_X ....A..... .......... .V........ .....A.... CS.....T.. ...S...... ..S...E... ..Q...GH.. .......... ..........

#Hosa_X EMILTDSNGE QPLSAMVSMV TKDNPGVVTC LDEARHGFES GDFVSFSEVQ GMVELNGNQP MEIKVLGPYT FSICDTSNFS DYIRGGIVSQ VKVPKKISFK
#Bota_X .......... .......... .......... .......... .......... ..I....S.. .......... .......... .......... ..........
#Mumu_Y .......... .........I ..E...I... .EDS...... ...I..T... ..S....IG. I......... .......S.. E......... ....R..N..
#Mumu_X ..V....... .......... .......... .........T .......... ..IQ...C.. .......... .......... .......... ..........
#Feca_Y ....S..S.. .......... ...C...... .......... ......T... ..N....TC. I......... .......... E..Q....R. ..........
#Feca_X .......... .......... ...S...... .N......G. .......... .......S.. .......... .......... .......... ..........
#Modo_X ......A... .......... .......... .......... ....T.T... ..S....IS. .......... ......AR.. ........T. ..........

#Hosa_X SLVASLAEPD FVVTDFAKFS RPAQLHIGF
#Bota_X ..P....... ..M.....Y. .........
#Mumu_Y P.L......E .........CC H........
#Mumu_X ..P...V... ..M.....Y. .........
#Feca_Y ..L....... ........Y. ..G......
#Feca_X ..L....... ..M.....Y. .........
#Modo_X ..PV.....E ..M....... ...H..VA.
```

H UBE1X/Y (UBE1X/Yb)

```
#Hosa_X MYMDRRCVYY RKPLLESGTL GTKGNVQVVI PFLTESYSSS QDPPEKSIPI CTLKNFPNAI EHTLQWARDE FEGLFKQPAE NVNQYLTDPK FVERTLRLAG
#Cafe_X .......... .......... .......... .......... .......... .......... .......... .......... .......... ..........
#Bota_X .......... .......... .......... .......... .......... .......... .......... .......... .......... ..........
#Mumu_X .......... .......... .......... .......... .......... .......... .......... .......... ........S. ..........
#Feca_X .......... .......... .......... .......... .......... .......... .......... .......... .......... ..........
#Modo_X .......... .......... .......... .......... .......... .......... .......... .......... S......... ..........
#Mumu_Y L.V....... .......... .........V .......... .......... .......... ...V...... .......S.. .......... .M....Q...
#Feca_Y ....C..... .......... .......... .......... .......... .......... .......... .......... ......I.S. ..........
#Maru_Y .....H.... .......... .....I.... .......... .......... .......... .......... ..S....... .......N.. ........G.
#Orna   Q.VAD...H. L..M.....Q ..R.SAG.YL ....QR.RAP VVNTSPTF.V ...RH..S.. .......... .....R.... T.HR..RE.S .L.TLEGAQA

#Hosa_X TQPLEVLEAV QRQRPQTWDC VTWACHHWHT QYSNNIRQLL HNFPPDQ
#Cafe_X .......... .......... .......... .......... .......
#Bota_X .......... .......... .......... .......... .......
#Mumu_X .......... .......... .......... ..C....... .......
#Feca_X .......... .......... .......... .......... .......
#Modo_X .......... .....RS... .A...L...A ..A....... .....E.
#Mumu_Y .........I HC........ ....YQ.... ...H..Q... .....A.
#Feca_Y .....M.... .......... ....Y....I .......... .....E.
#Maru_Y .......... H....HD... .....L...S ..A....... .....E.
#Orna   LTL..S.YSS LTH...D... .S..RRL.QL H.HDG..... LH...EK
```

I ATRX/Y

```
#Hosa_X EGGNLICCDF CHNAFCKKCI LRNLGRKELS TIMDENNQWY CYICHPEPLL DLVTACNSVF ENLEQLLQQN KKKIKVDSEH TRFSPKKTSS NCNGEEKKLD
#Cafa_X .......... .......... .......... .......... .......... .......... .......... .......ND. .......N.. ..........
#Bota_X .......... .......... .......... .......... .......... .......... .......... .......N.. A......N.. ..........
#Mumu_X .......... .......... .......... .......... ....Q..... .......... .......... ........DQ .K.....S.. S........E
#Loaf_X .......... .......... .......... .......... .......... .......... .......... ...M...ND. .......N.. ..........
#Modo_X .......... .......... .......... A......... ....R..... ......D... .......... ......E... SK.....NV. T.......V.
#Maeu_X .......... .......... .......... A.....S... ....R..... ......D... .......... ......E... .......NV. S.......S.
#Maeu_Y .........S .......... W.......I. K..N.K.E.H ....C..... ..IAV.D..L ...SH.K..K V.AAVKK.DC PE.HSSTKRE SNAE.KSQ..
#Orna   .......... .......... .......... A......... .......... ......D... .........S ...M..ENDA HK.....NNA ........FN

#Hosa_X DSCSGSVTYS YSALIVPKEM IKKAKKLIET TANMNSSRTN KSTGGEHKSD KEEQYEPAED LDMDIVSVPS SVPEDIFENL ETAMEVQSSV DHQEVESSVK
#Cafa_X .......... .......... .......... .T......A. ..VS...... .......... .......... .......... .........T .N..L.N...
#Bota_X ..Y....... ........D. .......... ........AH .NDST..... .K.....S.. .......... .......... .........A EY..L.T...
#Mumu_X E.....S... H...S..... ...TT..... .S......AH ...S...G.G .DG....T.. .......... .......DS. .S.......A .YP.L.....
#Loaf_X .A....I..A .......... .......... ........A. ...S....CG .KN.....D. .......... .......... .......G.A .F..L.....
#Modo_X ..H....... FT..M....I V..T...V.. .TS..A.PA. ..A.A.D.KS RK.RF....A .......... .........P .A.....V.S ENRD..H...
#Maeu_X .AY....... FT..M...DI V..T...V.. ..S..T.PVH .T.SA.D.KS RKDHF....A .......... .......... .A.......S ..RD..N...
#Maeu_Y N.Y..T.AH. F.S.NI.VDL ...TITVV.N ..V....KEI MVPRATTEVN N...N..PKS ....LP.F.. .R..V..K.V ..SVTI.NDE NTEVFKDTKC
#Orna   ..Y...I... .T..V...D. L......... .......AGS ...SR.D..N N........A .......... .......... .S......AA .FRVT..PT.

#Hosa_X LKDNRGGIKS KTTAKVTKEL YVKLTPVSLS NSPIKGADCQ EVPQDKDGYK SCGLNKQSET VDQNSDSDEM LAILKEVSRM SHSSSDTDIN EIHTNHKTLY
#Cafa_X .......... .......... .......... .......... .......... .S.......I .......... .......... .......... .T..HE....
#Bota_X .......... .......... .......... I......... .......S.. .SC......I ..HI...... .T...DMPS. .......... .P...K..F.
#Mumu_X ...S..N... .V....R... F......... ......V... ..S.E.N.R. .S.VA..PVI G......... ..V...A.Q. G......... .PQMHKGKTG
#Loaf_X ...S...... ..S....... .......... ...V...... ..S....V.. IS.......I .......... .......... .....ES... .T..HE...C
#Modo_X S..HK..T.F .IS....... .......... D..V.A.... .AT.E.EEN. .SVTS.PLD. .APS...E.I P.V....AM. .....TEQNE TNENPK.S..
#Maeu_X S..HK..T.F .I........ .......... D..V.A.... .AT.E.EES. .S..T.PL.. .AAS...... P.V....AML .....TEHKE TNENPK.S..
#Maeu_Y PESSEV.K.R PKLKTS.RK. ....S.I.K. DFSL.DTID. .KIE.EYQQE TSITGNTEVL KENIIIPGND TTL...ETDL QTTPLGQIEK .LVK.SEQSN
#Orna   SR.S..S..L .A........ .......... D..V.ATE.. D.S.E.EDDQ NPAAT.PL.A ...S...... P.V....AM. .....TDAHE TQGREKA.SG

#Hosa_X KDDKGKRKRK SSTSGSDFDT KKGKSAKSSI ISKKKRQTQS ESSNYDSELE KEIKSMSKIG AARTIPNTKD FDSSEDEKHS KKGMDNRLLR HKLTVSDGES
#Cafa_X .......... .......Y.I .......R.. .......N.. .......... .......... ....V..KE. Y........R .......... ..........
#Bota_X ....R..... .........I .....V.KA. .......N.. .......... ....R..... T...V..KEY Y......... .Q........ ....L.....
#Mumu_X ...N...... N......... .....TET.. .......NY. .......... R...T..R.. ...KV.EK.E E.......QG ..VV...... ....L.....
#Loaf_X .......... .........V .......... .......N.. .......... ....I..... .T..V..KE. Y......... ....A..... ..........
#Modo_X .G........ N.....E... ....AV..FT G.....HSH. .......... R...N.C... ..KQHS.KE. YG......QN A..SSK.... ...SG.....
#Maeu_X .GG.R..... T......... ....EV..FT S......NH. D......... R...T....V .KKPHSKKE. Y.......QI ....GK.... ...S......
#Maeu_Y SGQW..KG.. I.LPEEK.KL SNRE.NSD.S EKP..EAKN. SE.DT.LKD. NIQ.ELVSKA KCH.KAKR.R E..VTNLDND D.KSKAIKKQ QDENDFSSP.
#Orna   .S........ .........V Q..PAT.N.T TA.....NH. D......... R........R P.KKC.TKES L....E..Q. ...AGK.... ...SM.....

#Hosa_X GEEKTKPKES YKQKKKRRRI KVQEDSSSEN KSNSEEEESK KGRKKIRKID KLRTETQNAL KEEEERRKRI AERERERERKL REVIEIEDAP TKCPITTKLV
#Cafa_X ....M..... .......... .......... .......DK.. .......... .......... .......... .......... .......... ..........
#Bota_X ....M..... .......... .......... .......DK.. .......... .......... .......... .......... .......... ..........
#Mumu_X .......... .......... .......... ..H...DK.. .......... .......... .......... .......... .......... ..........
#Loaf_X ....M..... .......... .......... ..Q.YRMD.. .......... .......... .......... .......... .......... ..........
#Modo_X ....SR.... .......... .......... .......D.. .......... .......... .......... .......... .......... I.........
#Maeu_X ....AR.... .......... .......... .......D.. .......... .......... .......... .......... .......... I.........
#Maeu_Y SDMFSDKS.. .....RKHIK VKENL.TEH. .NL..ND... QS....KN.. ..QR...K.. ....R..... ..KQQ..... ...VDSSAS. .E....I...
#Orna   ....A..... .......... .......... .......D.. .......... .......... .......... .......... .......... V.........

#Hosa_X LDEDEETKEP LVQVHRNMVI KLKPHQVDGV QFMWDCCCES VKKTKKSPGS GCILAHCMGL GKTLQVVSFL HTVLKWQEGL KDDEKLEVSE LKRPQERSYM
#Cafa_X .......... .......... .......... .......... .......... .......... .......... .......... .......... ..........
#Bota_X .......... .......... .......... .......... .......... .......... .......... .......... .......... ..........
#Mumu_X ...N...... .......... .......... .......... .E........ .......... .......... .......... N.N....... ..........
#Loaf_X .......... .......... .......... .......... .......... .......... .......... .......... N......KV. ..........
#Modo_X .......... .........T .......... .......... .S.....A.. .......... .......... .......D.. D.E....... ..........
#Maeu_X .......... .........T .......... .......... .S.....A.. .......... .......... .......D.. D.E....... ..........
#Maeu_Y .......... ...I...L.T ......I... .......... .IQ....A.. .......... .......T.. .........V. E..K..K... ...A.D..DL
#Orna   .......... .........T .......... .......... .R.....T.. .......... .......... .......D.. E......... ..........

#Hosa_X IGYEMYRNLA QGRNVKSRKL KEIFNKALVD PGPDFVVCDE GHILKNEASA VSKAMNSIRS RRRIILTGTP LQNNLIEYHC MVNFIKENLL GSIKEFRNRF
#Cafa_X .......... .......... .......... .......... .......... .......... .......... .......... .......... ..........
#Bota_X .......... .......... .......... .......... .......... .......... .......... .......... .......... ..........
#Mumu_X .......... .......... .D........ .......... .......... ........K. .......... .......... .......... ..........
#Loaf_X .......... .......... .......... .......... .......... .......... .......... .......... .......... ..........
#Modo_X .......... .......... .......... .......... .......... .......... .......... .......... .......... ..........
#Maeu_X .......... .......... .......... .......... .......... .......... .......... .......... .......... ..........
#Maeu_Y .........V .........F ......T.I. .......... .......... .......... .......... .......... .......... ..........
#Orna   .......... .......... .......... .......... .......... .......... .......... .......... .......... ..........
```

```
#Hosa_X INPIQNGQCA DSTMVDVRVM KKRAHILYEM LAGCVQRKDY TALTKFLPPK HEYVLAVRMT SISIFRVYRF GQTKPVYVYR FLAQGTMEDK IYDRQVTKQS
#Cafa_X .......... .......... .......... .......... .......... .......... P......... .......... .......... ..........
#Bota_X .......... .......... .......... .......... .......... .......... P......... .......... .......... ..........
#Mumu_X .......... .......... .......... .......... .......... .......... A......... .......... .......... ..........
#Loaf_X .......... .......... .......... .......... .......... .......... P......... .......... .......... ..........
#Modo_X .......... .......... .......... .......... .......... .......... P......... ..N....... .......... ..........
#Maeu_X .......... .......... .......... .......... .......... .......... P......... ..N....... .......... ..........
#Maeu_Y .......... ..S.A...I. .......C.. .........H .T...I.... Y......... .V........ ..S...F... .......... ..E.......
#Orna   .......... .......... .......... .......... .......... .......... .......... ..N....... .......... ..........

#Hosa_X LSFRVVDQQQ VERHFTMNEL TELYTFEPDL LDDPNSEKKK KRDTPMLPKD TILLQIHKEH IV
#Cafa_X .......... .......... .......... .......... .......... .......... ..
#Bota_X .......... .......... .......... .......... .......... .......... ..
#Mumu_X .......... .......... .......... .......... .......... .......... ..
#Loaf_X .......... .......... .......... .......... .........V N..ALQKHMV PG
#Modo_X .......... .......... .......... .......... .......... .......... ..
#Maeu_X .......... .......... .......... .......... .......... .......... ..
#Maeu_Y .....I.... ......L... ....A..... ..VTDA.... .......... ....R....Y ..
#Orna   .......... .......... .......... .......... .......... .......... ..
```
