# Supplementary material for: No Evidence for a Second Evolutionary Stratum during the Early Evolution of Mammalian Sex Chromosomes
Source: PLoS One. 2012 Oct 19;7(10):e45488. doi: 10.1371/journal.pone.0045488 (PMC3477149; doi:10.1371/journal.pone.0045488)
Supplement: File S1 — The distribution and number of phylogenetically informative sites. We examined the distribution and number of phylogenetically informative sites by using only the second positions of codons, in which substitutions were unlikely to be saturated. This analysis excluded XKRX/Y, for which the Y homolog was not present in the opossum genome. For simplicity, 4 OTUs were used: the X and Y sequences from the opossum (marsupial X and Y: MX and MY) and a eutherian (human or cat), denoted by EX and EY (eutherian Y). Each phylogenetically informative site supports one of 3 possible topologies (Fig. S1). One topology (topology A: Fig. S1A) is supported by the partition as ([EX, MX], [EY, MY]), in which the inner parentheses indicate nucleotides that are shared. Gametologs that differentiated before therian divergence show the partition ([EX, MX], [EY, MY]), whereas differentiation after divergence produces the topology ([EX, EY], [MX, MY]). Furthermore, while the partition of ([EX, MY], [MX, EY]) is not consistent with early therian divergence, it could occur by chance (topology C: Supp Fig. S1 C). Table S2 shows the number of phylogenetically informative sites for each topology of the 4 genes (there are too few informative sites in HSFX/Y and RPS4X/Y to determine a topological category). The informative sites in SOX3/SRY and RBMX/Y support the differentiation of gametologs before therian divergence (Table S2), which is consistent with the topology of their nucleotide trees. SMCX/Y and UBE1X/Y support topology B (Fig. S2 B), suggesting differentiation after therian divergence (Table S2), which is not consistent with the topology of the nucleotide trees. (DOCX) [file pone.0045488.s007.docx]

**Supplementary methods**

We examined the distribution and number of phylogenetically informative sites by using only the second positions of codons, in which substitutions were unlikely to be saturated. This analysis excluded *XKRX/Y*, for which the Y homolog was not present in the opossum genome. For simplicity, 4 OTUs were used: the X and Y sequences from the opossum (marsupial X and Y: MX and MY) and a eutherian (human or cat), denoted by EX and EY (eutherian Y). Each phylogenetically informative site supports one of 3 possible topologies (Fig. S1). One topology (topology A: Fig. S1A) is supported by the partition as ([EX, MX], [EY, MY]), in which the inner parentheses indicate nucleotides that are shared. Gametologs that differentiated before therian divergence show the partition ([EX, MX], [EY, MY]), whereas differentiation after divergence produces the topology ([EX, EY], [MX, MY]). Furthermore, while the partition of ([EX, MY], [MX, EY]) is not consistent with early therian divergence, it could occur by chance (topology C: Supp Fig. S1*C*). Table S2 shows the number of phylogenetically informative sites for each topology of the 4 genes (there are too few informative sites in *HSFX/Y* and *RPS4X/Y* to determine a topological category). The informative sites in *SOX3*/*SRY* and *RBMX/Y* support the differentiation of gametologs before therian divergence (Table S2), which is consistent with the topology of their nucleotide trees. *SMCX/Y* and *UBE1X/Y* support topology B (Fig. S2*B*), suggesting differentiation after therian divergence (Table S2), which is not consistent with the topology of the nucleotide trees.
